# Prediction of allosteric sites and mediating interactions through bond-to-bond propensities

**DOI:** 10.1038/ncomms12477

**Published:** 2016-08-26

**Authors:** B. R. C. Amor, M. T. Schaub, S. N. Yaliraki, M. Barahona

**Affiliations:** 1 Department of Chemistry, Imperial College London, London SW7 2AZ, UK; 2 Institute of Chemical Biology, Imperial College London, London SW7 2AZ, UK; 3 Department of Mathematics, Imperial College London, London SW7 2AZ, UK

## Abstract

Allostery is a fundamental mechanism of biological regulation, in which binding of a molecule at a distant location affects the active site of a protein. Allosteric sites provide targets to fine-tune protein activity, yet we lack computational methodologies to predict them. Here we present an efficient graph-theoretical framework to reveal allosteric interactions (atoms and communication pathways strongly coupled to the active site) without *a priori* information of their location. Using an atomistic graph with energy-weighted covalent and weak bonds, we define a bond-to-bond propensity quantifying the non-local effect of instantaneous bond fluctuations propagating through the protein. Significant interactions are then identified using quantile regression. We exemplify our method with three biologically important proteins: caspase-1, CheY, and h-Ras, correctly predicting key allosteric interactions, whose significance is additionally confirmed against a reference set of 100 proteins. The almost-linear scaling of our method renders it suitable for high-throughput searches for candidate allosteric sites.

## Abstract (web summary)

Allostery is a key molecular mechanism underpinning control and modulation in a variety of cellular processes. Here, the authors present a method that can be used to predict allosteric sites and the mediating interactions that connect them to the active site of the protein.

## Graphical abstract

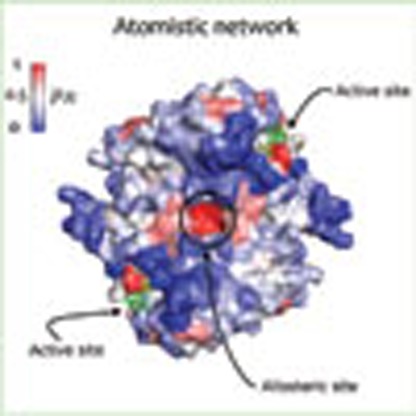

Allostery is a key molecular mechanism underpinning control and modulation in a variety of cellular processes^1^,^2^. Allosteric effects are those induced on the main functional site of a biomolecule by the binding of an effector at a distant site, for example, the binding of a cofactor modulating the catalytic rate of an enzyme^3^. Despite the importance of such processes, we still lack understanding as to how the interactions at the allosteric site propagate across the protein and affect the active site. Here, we present a graph-theoretic approach that uses atomistic structural data to identify the allosteric sites in proteins, as well as bonds and residues involved in signal propagation. Defining an edge-to-edge transfer function, we efficiently compute a bond propensity that captures the effect induced on any bond by perturbations at the active site. The resulting propensity score predicts allosteric sites and key bonds involved in mediating the allosteric propagation.

The realization that all proteins exhibit innate dynamic behaviour^4^,^5^ and the discovery of single-domain allosteric proteins^6^ have reaffirmed the ubiquity of allosteric regulation; potentially, any protein could be allosteric^7^. This fact has important experimental consequences: drugs targeted at allosteric sites could offer improved specificity compared with traditional active-site targets^3^. Efficient methods for identifying putative allosteric sites are therefore of great interest^8^. To date, computational approaches have involved statistical coupling analysis^9^, molecular dynamics^10^,^11^, machine learning^12^ and normal mode analysis^13^. For a comprehensive review see ref. 14.

Classic thermodynamic models of allostery, such as the Monod–Wyman–Changeux^15^ and Koshland–Némethy–Filmer models^16^, were formulated to explain cooperativity in multimeric proteins in terms of conformational transitions in the protein landscape^17^. Such models reproduce broad experimental features (for example, sigmoidal binding curves), but offer little insight into the molecular mechanisms driving the transition. In contrast, allosteric pathways aim to describe routes through which excitations propagate across a protein^9^,^18^,^19^. Recent experimental^20^,^21^ and computational^22^,^23^,^24^,^25^ work has showcased the anisotropy of energy flow in globular proteins, and linked anisotropy and allosteric behaviour^21^,^25^, for example, the anisotropic internal energy flow in albumin is altered by the binding of an allosteric ligand^21^. Our graph-theoretical calculations also reveal the anisotropy of the internal propagation of perturbations in proteins. However, we use the term ‘allosteric' specifically to describe distant locations where a perturbation can have a functional effect on the active site. The identification of such sites and the pathways connecting them to the active-site is an area of considerable interest^11^,^26^,^27^.

The connection between diffusion processes (for example, a random walk) on a network and the vibrational dynamics of the network is well established^28^,^29^. Previous network-based methods for protein structure analysis have used shortest-path calculations^30^, community-detection algorithms^31^ and random walks^32^. Such methods almost universally use ‘coarse-grained' residue–residue interaction networks (RRINs)^33^ without atomistic detail. Although obtaining edge weights for RRINs from molecular dynamics simulations yields improved results^34^,^35^, Ribeiro and Ortiz showed that RRINs are critically dependent on the chosen cutoff distance, and that energy-weighted networks including the covalent backbone are crucial for correctly identifying signal-propagation pathways^36^,^37^. Here, we show that exploiting the physico-chemical detail of atomistic, energy-weighted protein networks can enhance the identification of allosteric sites and mediating interactions.

We start by building an atomistic graph model of the protein: nodes are atoms, and weighted edges represent both covalent bonds as well as non-covalent bonds (hydrogen bonds, salt bridges, hydrophobic tethers and electrostatic interactions), with weights derived from interatomic potentials (see the section ‘Construction of the atomistic graph' and refs 38, 39). The resulting all-atom graph is analysed using the edge-to-edge transfer matrix *M*, a discrete Green's function in the edge space of the graph recently introduced in ref. 40 to study nonlocal coupling in graphs. Deriving an alternative interpretation of *M*, we show that it can be used to calculate the effect that the fluctuations of an edge have on any other edge of the graph. The resulting propensity score for each bond, Π_*b*_, measures how strongly bond *b* is coupled to the active site through the graph. This bond-to-bond formalism provides a natural way of uncovering how long-range correlations between bonds contribute to allosteric signalling. The computation time scales almost linearly in the number of edges^41^,^42^, making our method applicable to large systems with tens of thousands of atoms.

To establish if a bond has high propensity, we use quantile regression (QR)^43^, a robust statistical technique widely employed across fields^44^, to compare each bond to the ensemble of bonds within the protein at a similar geometric distance from the active-site. We also compare each bond propensity to a reference set of 100 representative proteins randomly drawn from the Structural Classification of Proteins (SCOP) database. This set provides a pre-computed structural bootstrap against which any protein can be tested in order to detect the statistically significant bonds, further reducing the computational cost.

We first analyse in detail three important allosteric proteins: caspase-1, CheY and h-Ras. In each case, given the location of the known active site, we correctly predict the location of the allosteric site and uncover communication pathways between the two sites. Each example highlights a particular aspect of the method. In caspase-1, comparison of our results with those obtained using RRINs shows that atomistic physico-chemical detail can be necessary for the reliable identification of the allosteric site. With CheY, we illustrate how information can be gained from ensembles of nuclear magnetic resonance (NMR) structures: the variance of the propensity across the NMR ensemble reveals residues involved in allosteric signalling that cannot be identified from the static X-ray structure alone. In h-Ras, we show that signal propagation between the active and allosteric sites is crucially dependent on the interaction between the protein and specific structural water molecules. Finally, we evaluate our approach against a further test set of 17 allosteric proteins. We find that the bond-to-bond propensity is a good predictor of allosteric potential, suggesting it could be used to guide efforts in structure-based allosteric drug discovery.

## Results

### Allosteric site and functional residues in caspase-1

**Figure 1:**
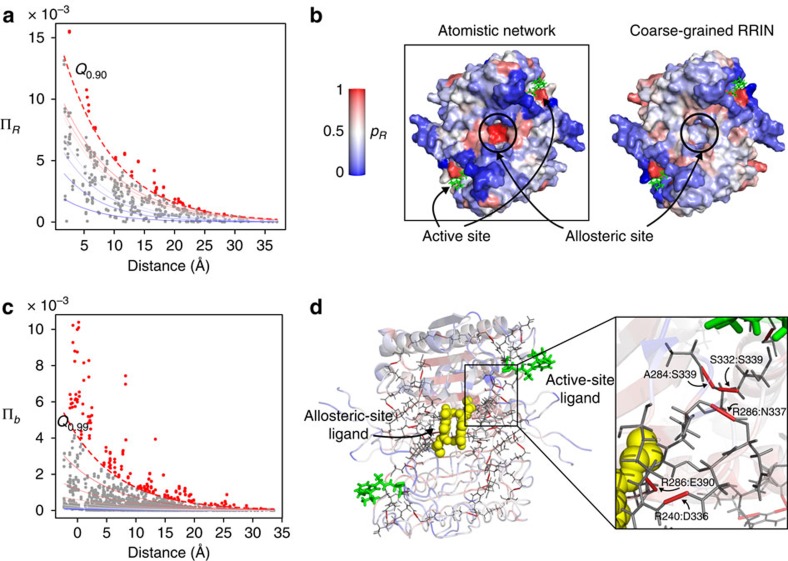
Bond-to-bond propensities identify the allosteric site and atomistic pathway in caspase-1. (**a**) The propensities of all residues Π_*R*_ are plotted against their distance from the active site. The lines correspond to the quantile regression estimates for the *p*-th quantiles *Q*_*p*_, with *p*=0.1,0.2,…,0.8,0.9. The dashed red line indicates the *Q*_0.90_ cutoff used for identifying important residues. (**b**) The quantile scores *p*_*R*_ for each residue are mapped onto the surface of caspase-1. The active-site ligand is shown in green. The allosteric binding site is identified as a hotspot of high propensity. When a coarse-grained RRIN with cutoff of 6 Å is used (right), the allosteric binding site is not identified. (**c**) The propensities of bonds Π_*b*_ are plotted against their distance from the active site with the *Q*_0.99_ quantile indicated by the dashed line. (**d**) High quantile score bonds (*p*_*b*_≥0.99) are shown on the structure. Bonds between R286:E390, R240:D336, R286:N337, A284:S332 and S332:S339 have large quantile scores and form contiguous pathways between the active and allosteric sites. The active-site ligand is shown in green and the allosteric ligand is shown as yellow spheres.

Our first example is caspase-1, an allosteric protein of importance in apoptotic processes^39^. Caspase-1 is a tetramer composed of two asymmetric dimers, each containing one active site. From the Protein Data Bank (PDB) atomic structure (2HBQ), we constructed an atomistic, energy-weighted graph representation of the protein based on interaction potentials, as described in ‘Construction of the atomistic graph'^38^,^39^. To quantify how strongly each bond is coupled to the active site, we calculate the propensities Π_*b*_ for all bonds in the protein (equation (8)), and we aggregate the bond propensities over each residue to obtain the residue score Π_*R*_ (equation (9)). We rank bonds and residues according to their significance by computing the corresponding quantile scores *p*_*b*_ and *p*_*R*_ obtained via QR, as given by equation (14). These quantile scores establish which bonds (residues) have high propensity values as compared with bonds (residues) at the same distance from the active site in the protein (Fig. 1a,c).

Our method finds a ‘hotspot' of residues with high quantile scores in a cavity at the dimer–dimer interface (Fig. 1b, left). This site has been previously identified by Scheer and co-workers. as the binding site for a small-molecule inhibitor of caspase-1 (ref. 45). Table 1 shows that residues within 3.5 Å of the allosteric inhibitor have significantly higher propensities than non-allosteric residues (Wilcoxon rank sum, *P*<0.0005). Residues E390, S332 and R286, which have been found to belong to a hydrogen bond network between the active and allosteric sites^45^, have respectively the third, 13th and 15th highest quantile scores of the 260 residues in each dimer of caspase-1.

Making use of the physico-chemical detail afforded by our atomistic description, we find the high propensity bonds that lie on communication pathways connecting the allosteric site to the active-site ligand. Concentrating on the top quantile *p*_*b*_≥0.99 (Fig. 1c), the two interactions between residues E390 and R286 have quantile scores of 0.996 and 0.990, and their combined propensity gives this salt bridge the highest quantile score in the protein. These salt bridges are directly disrupted by the allosteric inhibitor^45^. We also reveal other important bonds lying between the active and allosteric sites (Fig. 1d), including hydrogen bonds between Arg240:Asp336 (*p*_*b*_=0.999), S332:S339 (*p*_*b*_=0.996), R286:N337 (*p*_*b*_=0.992) and A284:S332 (*p*_*b*_=0.990). Bonds in this pathway have previously been identified by Datta *et al*.^45^ as being functionally important: the corresponding alanine mutations cause 230-fold (R286A), 130-fold (E390A), 3.7-fold (S332A) and 6.7-fold (S339A) reductions in catalytic efficiency.

The atomistic detail is important for the outcome of the analysis. If instead of employing an all-atom graph description, we carry out the same calculations on a coarse-grained RRIN^30^,^32^ with cutoff radius of 6 Å, the allosteric site of caspase-1 is no longer identified as a hotspot (Fig. 1b, right) and the allosteric residues do not have significantly higher propensity compared with other residues (Wilcoxon rank sum, *P*=0.5399). The results obtained with RRINs are in general dependent on the cutoff radius used. For caspase-1, the allosteric site is not detected in RRINs with cutoff radii of 6, 7 and 8 Å. The allosteric site is found to be significant with cutoff radius 10 Å, but the signal is considerably weaker than for the atomistic network (Supplementary Table 1). These findings highlight that while an atomistic model of the protein structure may not always be needed, it can indeed be important for the detection of allosteric effects in proteins. In this case, the strength of the pair of salt bridges formed by E390 and E286, which is crucial for the allosteric communication in caspase-1, is not captured by RRINs. Other recent results have similarly demonstrated the importance of both covalent bonds and hydrogen bonds to signal transmission within proteins^37^. Yet in other cases (for example, CheY in the following section), this level of physico-chemical detail seems to be less important, and RRINs are able to capture allosteric communication. An extended analysis of results for all-atom networks and RRINs with different cutoff radii for a variety of proteins can be found in Supplementary Note 1.

### Uncovering allosteric communication pathways in CheY

*Identifying the phosphorylation site of CheY*. CheY is a key protein in bacterial chemotaxis. When bound to the flagellar motor switch protein (FliM), it causes a change in the rotation direction of the flagellar motor, thus regulating the tumbling rate of *Escherichia coli*. This regulation is achieved through a post-translational modification; phosphorylation of CheY at the distant residue D57 increases its affinity for FliM, making this an interesting example of a single-domain allosteric protein.

**Figure 2:**
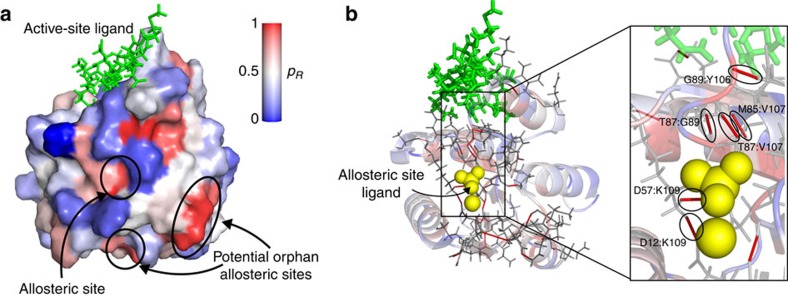
Allosteric phosphorylation site in CheY is identified by its high propensity. (**a**) Residue quantile scores *p*_*R*_ are mapped onto the surface of CheY. The allosteric phosphorylation residue D57 is identified as a hotspot. We identify two other distant sites, which could serve as potential orphan targets for allosteric effectors. (**b**) The top 3% of bonds by quantile score (that is, *p*_*b*_≥0.97) are indicated on the structure. The blow-up shows high quantile score non-covalent bonds that form propagation pathways between the allosteric ligand (yellow spheres) and the ligand-binding site (green).

We calculated the propensity of each bond and residue (relative to the FliM-binding site) in fully activated CheY (PDB ID: 1F4V) bound to Mg^2+^, BeF_3_ and FliM. We identify a number of hotspot surface residues with high quantile scores (Fig. 2a), including the phosphorylation site, D57 (*p*_*R*_=0.96). Residues in the allosteric site (<3.5 Å from phosphorylation site) have higher average quantile score than non-allosteric residues (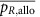
=0.61>
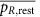
=0.43), and four of the seven residues in the allosteric site have high quantile scores, *p*_*R*_ ≥ 0.9 (Table 2). In addition, we find several previously unidentified distant surfaces with high quantile scores (Fig. 2a), which could correspond to putative (orphan) allosteric sites.

In contrast to caspase-1 above, using a RRIN with cutoff radius of 6 Å, we identify the phosphorylation site of CheY as a hotspot: the average quantile score of allosteric residues is much higher than for the rest of the residues (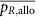
=0.72>
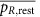
=0.46). Detection based on RRINs is robust over a range of cutoff radii 6–10 Å (Supplementary Table 1 and Supplementary Fig. 1). This result suggests that sometimes (for example, CheY) it is the topology of the protein structure that is important for signal propagation, whereas in other cases (for example, caspase-1) the specific atomistic structure given by the chemistry of the side-chain interactions matters for allosteric propagation. Our all-atom methodology incorporates both aspects consistently.

*Identifying allosteric communication networks*. Next, we examined allosteric pathways and bonds with high propensity in fully activated CheY (1F4V). Considering high quantile scores (*p*_*b*_≥0.97), we find several bonds connecting the allosteric phosphorylation site to the key binding site residue Y106 (Fig. 2b). One pathway comprises bonds between T87:E89 (*p*_*b*_=0.991) and E89:Y106 (*p*_*b*_=0.977); a second pathway is formed by K109, which has high quantile score bonds with D12 (*p*_*b*_=1) and D57 (*p*_*b*_=0.993). These residues have been discussed extensively in the biochemical literature as crucial for allosteric signalling (see Discussion).

**Figure 3:**
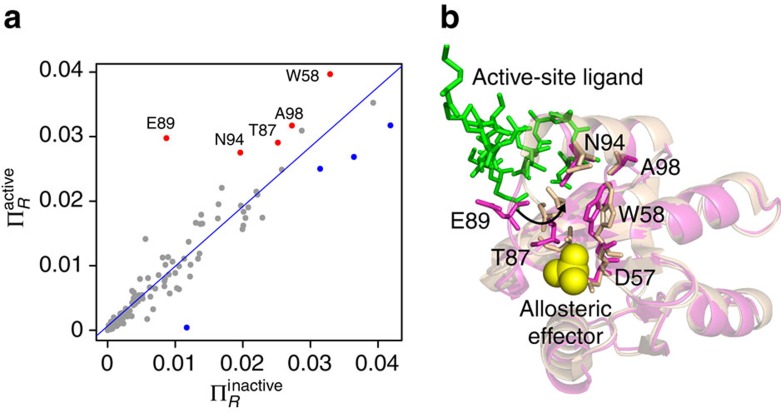
Comparison of residue propensities between active and inactive conformations of CheY. (**a**) The propensities most increased in the active X-ray structure (1F4V) as compared with the inactive X-ray structure (3CHY), as identified by Cook's distance, are coloured red and labelled. (**b**) Superposition of active (1F4V—beige) and inactive (3CHY—pink) conformations. The residues found in **a** form a pathway between the allosteric site and the ligand-binding surface.

In addition to fully activated CheY, we studied four conformations of CheY across a range of activation stages (details in Supplementary Table 2 and Supplementary Method 1). The profiles of bond-to-bond propensities are similar across all conformations (Supplementary Fig. 2), highlighting the robustness of the propensity scores to local dynamical rearrangements across different conformations. In particular, the propensities in the active (1F4V) and inactive (3CHY) conformations show a strong positive correlation (*r*=0.94). Using Cook's distance, a well-known method to detect influential points in linear regression^46^, we identify E89, N94, T87, A98 and W58 as residues with highly increased propensity in the active conformation as compared with the inactive conformation (Fig. 3a). Superposition of the active and inactive structures shows that the large displacement of E89 causes the formation of a tighter network of interactions involving N94, T87 and W58 in the active conformation (Fig. 3b). Interestingly, the propensity of the allosteric phosphorylation site D57 is similar in the active and inactive conformations; in the inactive conformation, D57 forms a strong hydrogen bond with K109, yet the weakening of this bond in the active conformation is compensated for by the formation of the network involving W58 and E89. Hence activation induces a structural rearrangement of the network of bonds that connect the phosphorylation site to the active site.

*Variability in NMR ensembles uncovers transient effects*. CheY exists in dynamic equilibrium between its active and inactive conformations, and X-ray structures have revealed an intermediate conformation with only the binding site adopting the active conformation^47^,^48^.

To explore the effect of small structural changes on the propensities of CheY, we analysed 20 NMR structures of the inactive conformation *apo*-CheY (PDB: 1CYE) and 27 NMR structures of the fully activated CheY bound to the phosphate mimic BeF_3_ (PDB: 1DJM). We calculated the average 〈Π_*R*_〉_NMR_ and the standard deviation SD(Π_*R*_)_NMR_ of the propensity of each residue over the ensemble of NMR structures, and compared them against the obtained from the X-ray structure.

The results of comparing NMR ensemble versus X-ray structures differ between inactive and active conformations, suggesting that dynamical reconfigurations have a consistent effect in the calculated propensities. For inactive CheY, the average ensemble NMR propensity of each residue, 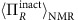, is strongly correlated (*r*^2^=0.96) with its X-ray propensity, 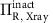, whereas for active Che-Y the correlation is weaker (*r*^2^=0.84), as seen in Supplementary Fig. 2. McDonald *et al*.^49^ have suggested that phosphorylation increases the flexibility of CheY, as reflected in increased B-factors and root-mean square fluctuations across the active NMR ensemble. Such enhanced flexibility may account for the greater difference in propensities between the NMR ensemble and X-ray structures for the active conformation.

**Figure 4:**
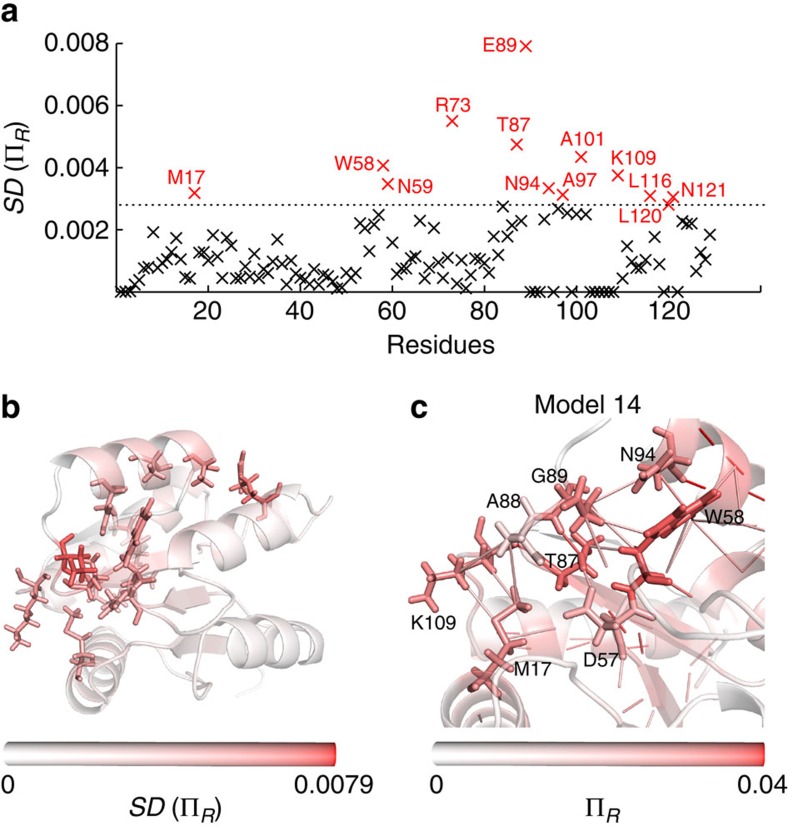
Increased variability of the propensity in NMR structures of active CheY reveals additional relevant residues. (**a**) Standard deviation of the residue propensities recorded over the NMR ensemble of 27 conformations corresponding to active CheY. The dashed line separates the top 10% of the residues by *SD *(Π_*R*_). Residue M17 has high NMR variability, although it was not identified in the X-ray structure as having high Π_*b*_. (**b**) The residues with high standard deviation are indicated on the structure, coloured by their NMR standard deviation. (**c**) Interactions coupling M17 to Y106 and the active site is shown in one of NMR conformations (model 14) of the active CheY. Residues coloured by their propensity Π_*R*_ in this particular conformation.

We computed the variability of the propensity of each residue across the active NMR ensemble (Fig. 4a). Among the residues with high (top 10%) NMR standard deviation SD(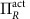)_NMR_, we find W58, T87, E89 and K109, which were also found to have high propensities in the active X-ray structure. These residues are known to be functionally relevant, and recent NMR relaxation–dispersion experiments have suggested that they form part of an allosteric network undergoing asynchronous local switching^49^. Other residues with high NMR s.d. are A101, R73, L116, K119 and N121. Of these, A101 lies in the α-helix forming the top half of the ligand-binding site, and the high variance of A101 and R73 can be explained by a hydrogen bond between these two residues transiently present across the active NMR ensemble. The other residues L116 and N121 lie in the α-helix forming the other side of the FliM-binding site: L116 forms a transient α-helical hydrogen bond with the ligand-binding residue K119, and N121 forms fluctuating hydrogen bonds with residues in, and adjacent to, the active site (Fig. 4b).

The large NMR variability of residue M17, which is 15 Å away from the active site, is of particular interest. CheY is intolerant to mutation of M17 (refs 50, 51), and it has been recently reported that this mutation causes chemical shift changes at Y106 (ref. 52), a key residue in the distant FliM-binding site. Our analysis shows that the propensity of M17 is higher in the active structure (both NMR and X-ray) than in the inactive structure: 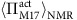
=0.0173>
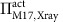
=0.0113>
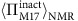
=0.0094>
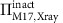
=0.0081. Furthermore, the NMR standard deviation of the propensity is higher in the active than in the inactive ensemble: *SD*
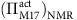
=0.0032>*SD*
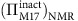
=0.0016. These results indicate that phosphorylation causes transient pathways to form between M17 and the active site that are not observed in the X-ray structure. By examining bonds with high propensity between M17 and Y106, we visually uncover a communication pathway involving residue K109 and three residues in the flexible *α*4—*β*4 loop: T87, A88 and E89. When we examine the individual NMR structure in which M17 has the highest propensity, M17 bonds directly with A88 and is indirectly connected to T87 through a hydrogen bond with K109 (Fig. 4c). This suggests that M17 is transiently coupled to Y106 through a network of hydrogen bonds and hydrophobic contacts not captured in the active X-ray structure. The transient making-and-breaking of particular bonds in the NMR ensemble translates into highly variable propensities associated with functionally important allosteric residues.

### Structural water is crucial to allosteric pathways in h-Ras

The enzyme h-Ras is a GTPase involved in signal transduction pertaining to cell cycle regulation^53^. Crystallographic evidence shows that calcium acetate acts as an allosteric activator in this process^54^. By comparing the calcium acetate-bound structure to the inactive structure, Buhrman *et al*.^54^ proposed a network of hydrogen bonds, involving structural water molecules, linking the allosteric site to the catalytic residue Q61.

**Figure 5:**
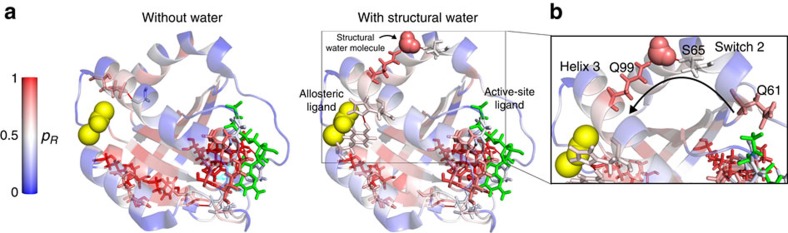
Structural water molecules are essential for the allosteric pathway in hRas. (**a**) Top percentile bonds by propensity quantile score (*p*_*b*_≥0.99) are shown on the structure: the left panel shows pathways identified without the inclusion of water molecules; and the right panel when structural water molecules are included in the graph. The structural water allows the formation of a pathway between the bottom of the switch 2 region and the top of helix 3, where the allosteric binding site is situated. The crucial water molecule which connects Q99 and S65 is indicated. (**b**) Blow-up indicating details of the pathway formed by Q99, a water molecule and S65, linking the allosteric pocket to the switch 2 region. The catalytic residue Q61 is shown at the bottom of switch 2.

We calculated the propensities and quantile scores of hRas bound to substrate and allosteric activator (PDB: 3K8Y) with and without inclusion of structural water molecules in the graph. In the absence of water (Fig. 5a, left), we find no bonds or residues with high quantile scores near the allosteric-binding pocket. When we include the eight molecules of structural water present in the PDB file, we identify a high quantile bond between the allosteric site residue Y137 and H94, and a pathway involving a structural water molecule that connects the allosteric region to a catalytic residue (Fig. 5b). Table 3 shows that the Q99-water and S65-water bonds involved in this pathway have the first and third highest quantile scores out of the 1159 weak interactions in the protein.

This water-mediated link between Q99 and S65 connects the allosteric binding pocket on helix 3 with the helical structure known as the switch 2 region, at the bottom of which lies the key catalytic residue Q61 (ref. 54). Our results suggest that structural water plays a crucial role in coupling the allosteric effector to the catalytic residue Q61.

### Absolute bond propensities against a SCOP reference set

**Figure 6:**
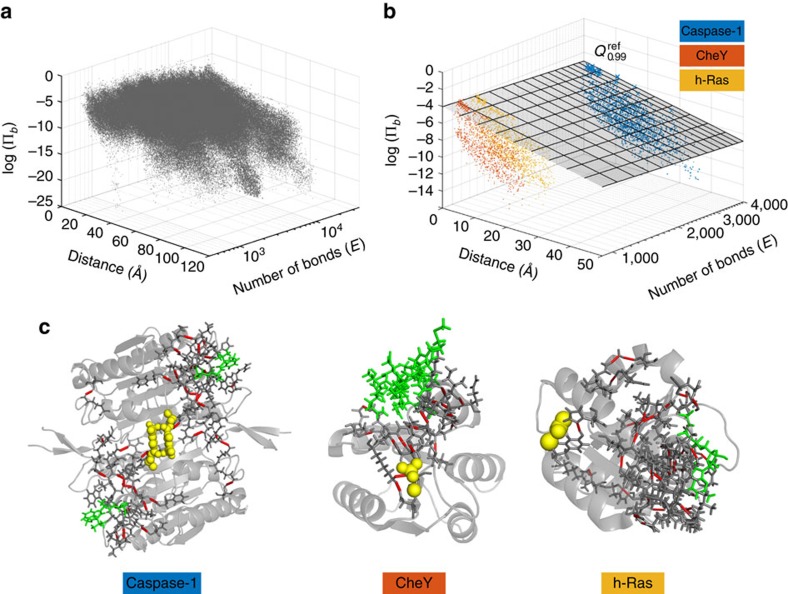
Calibration of absolute propensities against the SCOP reference set. (**a**) The logarithm of the bond propensity log(Π_*b*_) of all 465,409 weak bonds in the reference set (100 proteins from the SCOP database) plotted against *d*, the distance from their corresponding active site, and *E*, where *E* is the number of weak bonds in the corresponding protein. (**b**) The log propensities log(Π_*b*_) for caspase-1 (blue), CheY (orange) and h-Ras (yellow) are plotted together with the plane defining the 99th quantile fit obtained by solving the optimization equation (15) against the SCOP set of bonds shown in **a**. For each of the three proteins, there are bonds lying above the 99th quantile plane. (**c**) The bonds above the plane in **b** have 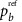
> 0.99 and are marked in red on the corresponding protein structures (active-site ligand in green, allosteric ligand as yellow spheres). The bonds thus identified play key allosteric roles, in agreement with the ‘intrinsic' results in previous sections.

The QR scores *p*_*b*_ in the previous sections identify bonds with high propensities compared with bonds at a similar geometric distance from the active site within the same protein. To assess the absolute significance of bond propensities, we assembled a reference set of 100 protein structures from the SCOP database^55^, and calculated the propensities (relative to the respective active sites) of all 465,409 weak bonds in this reference set (see Fig. 6a and Supplementary Method 2). Because the propensities are dependent on both the distance from the active site, *d*, and the total number of weak interactions in the protein, *E*, we apply QR against both *d* and *E*, as given by equation (15). The quantiles computed from the reference set can then be used to obtain absolute bond propensity scores (denoted 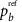) for any given protein without recomputing the regression.

We obtained the absolute quantiles 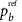 for the propensities of caspase-1, CheY and h-Ras studied above (Fig. 6b). Reassuringly, the significant bonds are also found to be important according to the absolute measure, with a strong correlation between propensity scores and absolute propensity scores (Supplementary Fig. 4). Visualization of the bonds with high absolute scores (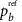
≥ 0.99) show they form pathways between the active and allosteric sites (Fig. 6c). These results confirm that the importance of these bonds not only relative to other bonds within the respective protein, but also in absolute terms relative to the protein reference set.

### Validating the propensity measure on an allosteric test set

To test our methodology, we computed the bond propensities of 17 additional proteins known to exhibit allostery. Ten of these proteins were taken from a benchmark set collected by Daily *et al*.^56^ and a further seven were obtained through an extensive literature search. (Five proteins in ref. 56 could not be used due to the presence of non-standard amino-acids, to the absence of an allosteric ligand, or to a mismatch between the oligomeric state of the active and inactive structures.) For details and structures of all 20 proteins analysed in the paper, see Supplementary Table 3 and Supplementary Fig. 5.

**Figure 7:**
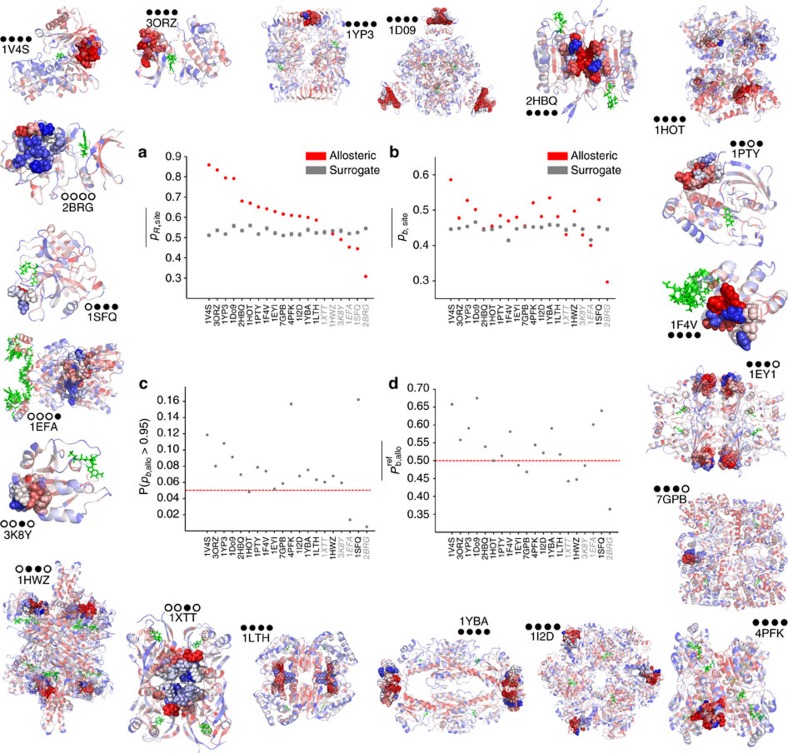
Prediction of allosteric sites based on bond-to-bond propensity for a test set of 20 allosteric proteins. The structures of the 20 proteins in the test set (labelled by PDB code) have their residues coloured by their quantile score *p*_*R*_, and the allosteric site is shown as spheres. For full details of these proteins, see Supplementary Table 2. The four statistics computed from our propensity are showed in the centre: (**a**) average residue quantile scores in the allosteric site 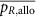 (red) compared with the average score of 1,000 surrogate sites 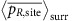 (grey), with a 95% confidence interval for the average from a bootstrap with 10,000 resamples (see ‘Structural bootstrapping'); (**b**) average ‘bond' quantile scores in the allosteric site against the equivalent bootstrap of 1,000 surrogate sites; and (**c**) tail of the distribution of bond propensities, that is, proportion of allosteric site bonds with quantile scores *p*_*b*,allo_>0.95. Proteins above the expected proportion of 0.05 (red line) have a larger than expected number of bonds with high quantile scores; (**d**) average ‘reference' bond quantile score in the allosteric site 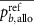. The red dotted line indicates the expected value of 0.5, and proteins above this line have a higher than expected reference quantile score. For the numerical values of all measures see Supplementary Table 3. The four circle code by each protein indicates whether the allosteric site is identified (filled circle) or not identified (open circle) according to each of the four measures (**a**–**d**). Nineteen out 20 allosteric sites are identified by at least one measure, and 15 out of 20 sites are identified by at least three of four measures.

For each protein, we calculate the propensity quantile scores (with respect to their active site) of all its bonds and residues, both intrinsic (*p*_*b*_, *p*_*R*_) and absolute (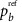). No *a priori* knowledge about the allosteric site was used. Figure 7 shows the 20 protein structures coloured according to the residue quantile score *p*_*R*_, with the allosteric sites marked with spheres. To validate our findings on this test set, we used the location of the allosteric site *a posteriori* and evaluated the significance of the computed allosteric quantile scores according to four statistical measures (Fig. 7a–d). See ‘Statistical evaluation of allosteric site quantile scores' for a full description and definitions.

The allosteric site is detected significantly by at least one of the four measures in 19 out of 20 proteins in the test set, and is detected by three or more of the four measures in 15 out of 20 proteins in the test set. The full numerical values are given in Supplementary Table 4. In practice, all statistical measures provide important and complementary information about the distribution of bond propensities, and can be used conjointly for the robust detection of allosteric sites.

## Discussion

Using protein structural data to construct an atomistic energy-weighted network with covalent and non-covalent bonds, we have defined a graph-theoretic measure of bond-to-bond propensity and used it to identify allosteric sites without prior information as to their location. Our propensity measure identifies bonds that are strongly coupled to the active site via communication pathways on the protein graph, even if they are separated by large geometric distances. Allosteric sites correspond to ‘hotspots', that is, sites with high propensity to perturbations at the active site as measured by their quantile score relative to other sites in the protein at a similar distance from the active site. This finding suggests that the structural features embedded in the architecture of the protein are exploited to enhance the propagation of perturbations over long distances.

Comparing against a representative reference set of 100 proteins randomly assembled from the SCOP database, we computed absolute quantile scores to further confirm the significance of bond propensities. One advantage of this absolute measure is that the QR over the reference set need not be recalculated, and the absolute bond quantile scores of any protein of interest can be obtained directly against them, thus further reducing the analysis time.

We have validated our method on a test set of 20 allosteric proteins without using any *a priori* information about their allosteric sites. We used our quantile scores and a structural bootstrap to define four statistical measures of significance based on the average and tail of the distribution of bond propensities in the allosteric site. The allosteric site is detected for 19/20 proteins, according to at least one statistical measure, and for 15/20, according to at least three of four statistical measures. These findings indicate the robustness of bond propensity as a predictor of allosteric sites and its potential to guide structure-based drug discovery efforts, for example, by ranking putative binding sites based on their allosteric potential. Our method also uncovers hotspots not previously identified as allosteric sites (see CheY in Fig. 2). Hardy and Wells^8^ have discussed the existence of ‘orphan' or ‘serendipitous' allosteric sites targeted by as-yet undiscovered natural effectors or open for exploitation by novel small molecules. The identified sites could provide targets for mutational analysis or allosteric small-molecule inhibition.

We have exemplified our method with a detailed analysis of three proteins (caspase-1, CheY and h-Ras), focussing on the contribution of high propensity bonds to pathways (or networks) of weak bonds linking the active and allosteric sites. The weak bond network found in caspase-1 (E390/R286/S332/S339/N337) has previously been tested experimentally and shown to be functionally important^45^. In CheY, we found that bonds between T87:E89 and E89:Y106, with very high quantile scores, are key to a transmission pathway for the signal induced by phosphorylation, also consistent with experimental evidence^47^,^49^,^57^. We also found a second pathway in CheY involving the bond K109:D57 (third highest quantile score). Interestingly, mutation of K109 abolishes chemotactic activity^50^ and has been proposed to form part of the post-phosphorylation activation mechanism^58^. Comparison of bond propensities across active/inactive conformations and across NMR data further confirmed K109 as a central link in the communication between the phosphorylation and binding sites in CheY.

Determination of protein structures from NMR solution experiments results in multiple models, each consistent with experimentally derived distance restraints. The ensemble of structures is not a true thermodynamic ensemble, since variation could be due to actual flexibility and thermal motion during the experiment, or to inadequate (or under-constrained) interatomic distance restraints. Our analysis suggests that the variation within NMR structures can reveal functionally relevant information. For CheY, residues with highly variable propensities across the NMR ensemble (E89/W58/T87/E89/K109) form an asynchronously switching allosteric circuit after phosphorylation, as revealed by NMR relaxation–dispersion experiments^49^. We also identify residue M17 as having high propensity in the NMR ensemble due to a transient network of interactions. This may explain experiments showing that mutation of M17 has a functional effect and induces chemical shift changes at Y106 (ref. 52).

Comparison across conformations indicates that propensities are fairly robust to local dynamic fluctuations, as shown by the strong correlation between active and inactive conformations and across NMR structures (Fig. 3 and Supplementary Figs 2 and 3). Additionally, we show in Supplementary Note 2 and Supplementary Tables 5 and 6 that the propensities, and the identification of significant residues and bonds, are generally robust to both randomness in the bond energies and to the breakage of a large proportion of weak interactions. On the other hand, as discussed above, further information about residues and bonds can be obtained by evaluating the highest variations induced by dynamical and structural variations. A fuller investigation of the effect of dynamics on the calculated propensities using experimental data (NMR conformations) and molecular dynamics simulations would thus be an interesting area for future research.

The role of structural water molecules in mediating allosteric communication has so far received limited attention. In a recent study of a PDZ domain, Buchli *et al*.^59^ suggest that changes in water structure could mediate communication with remote parts of the protein. Our analysis of h-Ras found that including structural water molecules was necessary to reveal a pathway linking the allosteric and active sites. These results suggest that novel methods to study interaction networks between proteins and water deserve further investigation. The addition of bulk water would require the simulation of hydration, including energy minimisation and equilibration steps, but the computational efficiency of our method would make it possible to analyse all-atom representations of such hydrated structures.

To what extent does the identification of the allosteric site require an atomistic, chemically detailed graph construction? To answer this question, we applied our propensity measure to RRINs, the coarse-grained residue-level models used in almost all previous network analyses of proteins. For caspase-1, we found that allosteric residues are not significant in RRINs (across several different cutoff radii), whereas, on the other hand, the allosteric site of CheY was consistently detected by both atomistic and residue-level descriptions. This indicates that both coarse topological features and detailed chemical communication pathways can be relevant for allostery, depending on the protein. Hence the atomistic graph with detailed physico-chemical information can in some cases be important to capture the communication features of the protein, for example, in caspase-1, the binding of the allosteric ligand perturbs a network of strong hydrogen bonds and salt bridges as identified in our analysis. The analysis of RRINs for all 20 proteins in our allosteric test set (Supplementary Note 1) confirms that the outcome varies by protein and can also be dependent on the choice of cutoff radius^37^. We emphasise, however, that our propensity measure is agnostic to the network model under analysis, allowing for the evaluation of distinct graph-construction techniques (for example, atomistic versus coarse-grained) and the use of different force fields.

Finally, it is important to remark that our method is computationally efficient. To obtain the bond-to-bond propensities, we solve a sparse linear system (equation (6)) involving the (weighted) Laplacian of the protein graph. As discussed in ‘Computational cost of bond-to-bond propensity', recent algorithmic advances allow us to solve such linear systems in almost linear time^41^,^42^. Hence protein complexes of ∼100,000 atoms can be run in minutes on a standard desktop computer. We can thus maintain atomistic detail, yet analyse large biomolecular complexes that are intractable for traditional computational methods.

## Methods

### Mathematical derivation of the bond-to-bond propensity

*Fluctuations and the edge-to-edge transfer matrix of a graph*. The edge-to-edge transfer matrix *M* was introduced in ref. 40 as a nonlocal edge-coupling matrix for the analysis of weighted undirected graphs, based on the concept of flow redistribution. It was shown there that the element *M*_*ji*_ reflects the effect that an injected flux on edge *i* has on the flux along edge *j* after the fluxes are redistributed over the whole graph when at equilibrium. Alternatively, *M* can be understood as a discrete Green's function in the edge space of the graph. See ref. 40 for detailed derivations and applications.

Here, we derive a complementary interpretation of the edge-to-edge transfer matrix *M*, which can be understood as describing how fluctuations of edge weights propagate through the graph. This reinterpretation underpins the work in this paper, linking *M* to the analysis of bond fluctuations in biomolecules.

As a starting point, consider the well-known Langevin equation, also denoted the heat kernel equation^60^,^61^:


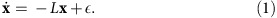


Formally, equation (1) has the same structure as the canonical model for scalar vibrations with nearest neighbour interactions encoded by the matrix *L*^28^,^29^. Alternatively, equation (1) may be considered as a model of a diffusing particle transitioning like a random walker on the underlying graph structure represented by *L*. In contrast to coarse-grained methods^32^, the variable **x** here is associated with atomic fluctuations, that is, our graph model reflects an atomic description that incorporates physico-chemical interactions derived from the three-dimensional structure of the protein in the PDB file. The resulting graph contains energy-weighted interactions representing bonds in the protein, including both covalent bonds and weak interactions such as hydrogen bonds, salt bridges, hydrophobic tethers and electrostatic interactions. For details of the graph construction see ‘Construction of the atomistic graph' and Supplementary Method 4.

The matrix *L* is the graph Laplacian^62^:


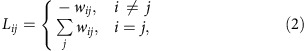


where *w*_*ij*_ is the weight of the edge between nodes (atoms) *i*,*j*. In this case, *w*_*ij*_ is the energy of the bond between both atoms. Thermal background fluctuations are modelled by ϵ, a zero mean white Gaussian noise input vector, that is, a simple heat bath acting independently on all atomic sites with covariance matrix


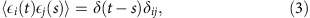


where *δ* stands for the Dirac delta function.

Instead of focusing on the atomic (node) variables **x**, we wish to study the coupling between bonds, and thus concentrate on the bond (edge) variables of the graph:


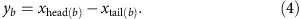


Clearly, *y*_*b*_ describes the difference of the node variables at the endpoints of the associated bond *b*, that is, a fluctuation associated with the bond between two atoms. The vector of bond fluctuations can be compactly represented in vector notation as


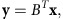


where *B* is the incidence matrix of the graph relating each edge variable to its corresponding node variables, that is, *B*_*bi*_=1 if node *i* is the head of bond *b*; *B*_*bi*_=−1 if node *i* is the tail of bond *b*; and *B*_*bi*_=0 otherwise.

We can now calculate the cross-correlations between edge fluctuations as


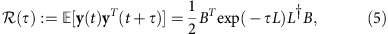


where *L*^†^ is the (Moore–Penrose) pseudo-inverse of the Laplacian matrix. Each entry 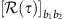 describes how a fluctuation at bond *b*_2_ is correlated with a fluctuation at bond *b*_1_ at time *τ*. See Supplementary Note 3 for a full derivation of equation (5).

Biophysically, we are ultimately interested in the energy fluctuations induced by bonds on other bonds. Therefore, we multiply the correlation matrix 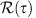 by the diagonal matrix of bond energies, *G*=diag(*w*_*b*_):


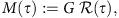


to obtain the matrix of bond-to-bond energy correlations with delay *τ*. Our measure of bond-to-bond propensity is obtained from the instantaneous correlations (that is, *τ*=0) leading to the edge-to-edge transfer matrix:


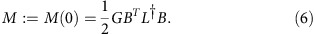


Note that the diagonal entries of *M* are indeed related to the average energy stored in the bond fluctuations: 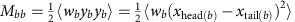. Likewise, the off-diagonal entries *M*_*b*1*b*2_ reflect how a perturbation at bond *b*_2_ affects another bond *b*_1_ weighted by the strength of bond *b*_1_. Hence the influence on a stronger bond is considered to be more important. Although we have not considered here time-delayed correlations (that is, as a function of *τ*), this is an interesting direction for future research.

*Definition of the bond-to-bond propensity*. To construct our measure of propensity, we only assume knowledge of the active site and proceed as follows. Let us consider all the ligand–protein interactions formed at the active site and compute their combined effect on each bond *b* outside of the active site:


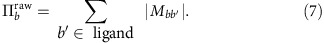


This raw propensity reflects how closely the active-site is coupled to each individual bond. Note that the computations include all the bonds in the protein (covalent and non-covalent). However, in the paper we only report the effect on weak bonds, since it is changes in weak-bonding patterns that usually drive allosteric response in proteins. Since different proteins have different numbers of bonds, we make the measure consistent by normalizing the score:


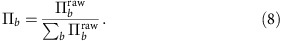


Throughout the manuscript, the quantity Π_*b*_ is referred to as the propensity of bond *b*; a measure of how much edge *b* is affected by the interactions at the active site. The propensity of a residue is defined as the sum of the (normalized) propensities of its bonds:


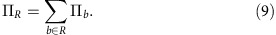


*Computational cost of bond-to-bond propensity*. The computation of the propensities is efficient. Note that equation (8) requires the summation over columns of the *M* matrix corresponding to protein–ligand interactions. Crucially, we do not need to compute the full pseudo-inverse *L*^†^ in equation (6); we can instead solve a sparse linear system involving the graph Laplacian. Recent algorithmic developments^41^,^42^ have made this possible in almost linear time, 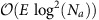, where *E* is the number of bonds (edges) and *N*_*a*_ is the number of atoms (nodes). Our method therefore is scalable to large systems. Using the Combinatorial Multigrid toolbox written by Koutis^63^ (available at http://www.cs.cmu.edu/jkoutis/cmg.html) propensities for all the bonds in proteins with ∼100,000 atoms can be run in minutes on a standard desktop computer.

### Significance of propensities through quantile scores

To identify bonds (and residues) with high propensities relative to others at a similar distance from the active site, we use quantile regression^43^, a technique of wide use in econometrics, ecology and medical statistics. In contrast to standard least squares regression, which focusses on estimating a model for the conditional mean of the samples, QR provides a method to estimate models for conditional quantile functions. This is important for two reasons: (i) the conditional distributions of propensities are highly non-normal; and (ii) we are interested not in the average bond, but in those bonds with particularly high propensities lying in the tails of the distribution. Once the fitted models are obtained, the quantile score of a bond *p*_*b*_ is a measure of how high the propensity Π_*b*_ is relative to other bonds in the sample which are at a similar distance from the active site.

Although QR goes back more than 200 years, it has only become widely used recently, due to the availability of computational resources. The mathematical basis of the method stems from the fact the *p*^th^ quantile, *Q*_*p*_, of a distribution is given by the solution of the following optimization problem: given a sample {*y*_*i*_}_*i=*1_^*n*^ parametrically dependent on *m* variables 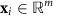 with parameters *β*, the estimate of the conditional *p*^th^ quantile of the sample distribution is obtained by solving


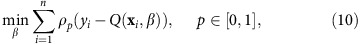


where *ρ*_*p*_(·) is the tilted absolute value function


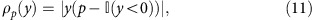


and *I*(·) is the indicator function. If the dependence is assumed to be linear, *Q*(**x**_*i*_,*β*)=*β*_0_+*β*^*T*^**x**_*i*_, the optimization can be formulated as a linear program and solved efficiently through the simplex method to obtain 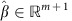, the estimated parameters defining the model^43^.

In the sections ‘Allosteric site and functional residues in caspase-1', ‘Uncovering allosteric communication pathways in CheY' and ‘Structural water is crucial to allosteric pathways in h-Ras', we have applied QR to the propensities Π_*b*_ of bonds within each protein so as to take into account their dependence with respect to *d*_*b*_, the minimum distance between bond *b* and any bond in the active site:


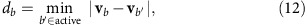


where the vector **v**_*b*_ contains the coordinates of the midpoint of bond *b*. On the basis of the observed exponential decay of Π with *d*, we adopt a linear model for the logarithm of the propensities and estimate the conditional quantile functions by solving the minimization problem


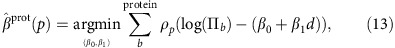


where the sum runs over the weak bonds of the corresponding protein. From the estimated model for the protein, we then calculate the quantile score of bond *b* at distance *d*_*b*_ from the active site and with propensity Π_*b*_, by finding the quantile *p*_*b*_, such that


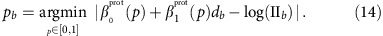


Similarly, in ‘Absolute bond propensities against a SCOP reference set', we use QR to obtain absolute quantile scores of bonds and residues with respect to a reference set of 100 proteins from the SCOP database. In this case, the propensities are regressed against both the distance to the active site *d*, and the number of non-covalent bonds in the protein, *E*. Since the mean propensity scales as *E*^−1^, we also assume a power-law dependency of the quantiles. Hence, we solve


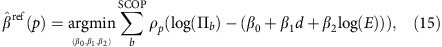


where the sum runs over all the weak bonds of all the proteins in the SCOP reference set. For each quantile *p*, the model is defined by the equation of a plane 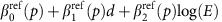 (Fig. 6b). The global quantile score 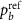 for bond *b* at a distance *d*_*b*_ from the active site in a protein with *E*_*b*_ non-covalent bonds is found by solving


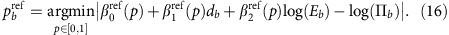


Quantile scores for residues are obtained by applying the same process to the propensities Π_*R*_.

The QR computations have been carried out using the R toolbox *quantreg* (http://cran.r-project.org/web/packages/quantreg/index.html) developed by Koenker^64^.

### The SCOP reference set of generic proteins

The SCOP database is a manually curated database which uses a hierarchical classification scheme collecting protein domains into structurally similar groups^55^. The major classes of cytoplasmic proteins in the database are *α*, *β*, *α*/*β*, *α*+*β*, and multi-domain, covering all the major fold-types for cytosolic proteins. To obtain a representative set of proteins from the database, we randomly selected 20 proteins from each of the five classes. Note that we only include proteins for which there is a structure with a ligand bound to the active site. Our reference set thus covers a broad region of protein structure space. Details of the 100 proteins selected can be found in Supplementary Method 2.

For each protein in the data set, we compute the distance from the active site, *d*_*b*_, and we calculate the propensity, Π_*b*_, for all its *E* weak bonds. Across the 100 proteins, we obtain a total of 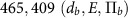 triplets corresponding to all the weak bonds in the proteins of the reference set (Fig. 6a). We then use QR to fit quantiles to this reference set, as given by equation (15). Note that the estimated quantile models, which are conditional on *d* and *E*, are now referred to the whole SCOP reference set and are not specific to any one particular protein. We then use the quantiles of the reference set to compare the bond propensities of any protein of interest and compute the ‘absolute' quantile score *p*_*b*_^ref^ for each bond, as given by equation (16). This score measures how high the bond propensity is, given its distance from the active site and the number of weak bonds in the protein of interest, as compared with all the bonds contained in the wide range of proteins represented in the SCOP reference set.

### Statistical evaluation of allosteric site quantile scores

To validate our findings on the allosteric protein test set, we evaluated the significance of the computed quantile scores according to four statistical measures, based on the following metrics:

(i) The average bond quantile score:


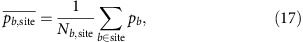


where *N*_*b*,site_ is the number of bonds in the site.

(ii) The average residue quantile score:


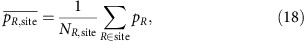


where *N*_*R*,site_ is the number of bonds in the site.

(iii) The proportion of allosteric bonds with *p*_*b*_ > 0.95, denoted P(*p*_*b*,allo_ > 0.95). Since the quantile scores are uniformly distributed, 0.05 is the expected proportion of bonds with quantile scores above 0.95.

(iv) The average reference bond quantile score:


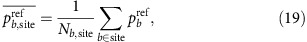


where *N*_*b*,site_ is the number of bonds in the site.

These four measures are introduced to check robustly for the significance of the bonds in the allosteric site from distinct perspectives. If the functional coupling between active and allosteric sites is due to a cumulative effect of the entire allosteric site, then average quantile scores over all bonds in the allosteric site should be an accurate measure of its allosteric propensity. Measures (i), (ii) and (iv) capture this property at the level of bonds and residues for both intrinsic and absolute propensities. It is also possible that functional coupling to the active site is concentrated on a small number of high quantile score bonds, with most others only being involved in structural or energetic aspects of binding to the allosteric ligand and having low quantile scores. Our metric (iii), which measures the number of high quantile score bonds in the site, can capture this behaviour based on the tail of the distribution. Reassuringly, the four measures provide complementary, yet largely consistent outcomes.

*Structural bootstrapping*. To establish the significance of the average quantile scores 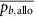 and 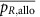, we assess them against random surrogate sites sampled from the same protein, used as a structural bootstrap. The surrogate sites generated satisfy two structural constraints: (1) they have the same number of residues as the allosteric site; (2) their diameter (that is, the maximum distance between any two atoms in the site) is not larger than that of the allosteric site. The algorithm for generating these sites is described in Supplementary Method 3. For each protein, we generate 1,000 surrogate sites and calculate their quantile scores 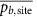 and 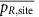. The average scores over the ensemble of 1,000 surrogate sites 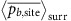 and 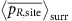, where the angle brackets denote the ensemble average, are then compared against the average residue quantile score of the allosteric site (Fig. 7a,b). A bootstrap with 10,000 resamples with replacement^65^ was used to obtain 95% confidence intervals providing statistical signficance.

*Validation on the allosteric test set*. Figure 7a–d reports these four statistical measures for all 20 proteins analysed (see Supplementary Table 4 for the corresponding numerical data). Our results indicate robust identification of the allosteric sites in the test set. The quantile score of the allosteric site is higher than that of the surrogate sites and above the 95% bootstrapped confidence interval in 14 out of 20 proteins for the residue score, 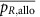, and for 16 out of 20 proteins for the bond score, 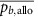 (Fig. 7a,b). The proteins identified by both measures are almost coincident, with few differences: Glutamate DH (1HWZ) is significant according to the bond score and marginally below significance according to the residue score, whereas the opposite applies to Thrombin (1SFQ). The reason for these differences lies with the distribution of bond scores: in some cases, allosteric sites have only a few bonds with high quantile scores and many other less important bonds. When considered at the level of residues, this can lead to high *p*_*R*_ scores; yet when bonds are considered individually through their *p*_*b*_ scores, the high quantile scores are averaged out over the whole allosteric site.

To evaluate the presence of high scoring bonds, we compute the proportion of bonds with high quantile score P(*p*_*b*,allo_>0.95) in the allosteric site, as compared with the expected proportion (0.05) above this quantile. The proportion of high quantile score bonds in the allosteric site is greater than expected in 17 of the 20 proteins (Fig. 7c). Of these 17 proteins, 16 coincide with those identified using the average scores reported above, and we additionally identify h-Ras (3K8Y). This finding confirms that allosteric sites consistently exhibit a larger than expected number of bonds with a strong coupling to the active site.

Finally, we compute the average absolute quantile score of the allosteric site 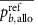 against the SCOP reference set (Fig. 7d). The results are largely consistent with the intrinsic measure 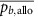: in 14/20 proteins, the absolute quantile score is greater than the expected 0.5, that is, 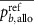
>0.5. Yet some proteins (for example, glutamate dehyrogenase (1HWZ), fructose 1,6-bisphosphatase (1EYI), and glycogen phosphorylase (7GPB)) have high intrinsic quantile scores, as compared with other bonds in the same protein, but do not score highly in absolute value, as compared with the reference SCOP ensemble. This result highlights the fact that a site need not have a high absolute propensity, as long as its propensity is high in comparison with the rest of the protein it belongs to, so that the ‘signal' from the site outweighs the ‘noise' from the rest of the protein. Interestingly, the lac repressor (1EFA) has an allosteric site with large absolute propensity (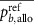
=0.60>0.5) but non-significant intrinsic propensity.

### Construction of the atomistic graph

An in-depth discussion of the construction of the graph can be found in refs 38, 39, and further details are given in Supplementary Method 4. Briefly, we use an atomistic graph representation of a protein, where each node corresponds to an atom and the edges represent both covalent and non-covalent interactions, weighted by bond energies derived from detailed atomic potentials. The covalent bond energies are taken from standard bond dissociation energy tables. Non-covalent interactions include hydrogen bonds, salt bridges, hydrophobic tethers and electrostatic interactions. Hydrogen bond energies are obtained from the DREIDING force-field^66^. Attractive hydrophobic interaction energies are defined between carbon and sulphur atoms, according to a hydrophobic potential of mean force introduced by Lin *et al*.^67^. Electrostatic interactions with coordination ions and ligands are identified from the LINK entries in the PDB file, with bond energies assigned using a Coulomb potential.

To compare the results between our atomistic model and residue-level RRINs^32^, we use coarse-grained network models obtained from the oGNM server^68^. A detailed comparison of results obtained with atomistic networks and RRINs is given in the Supplementary Note 1. We note that the main methodology (that is, the propensity measure and methods developed in the sections ‘Mathematical derivation of the bond-to-bond propensity' and ‘Significance of propensities through quantile scores') is independent of the construction of the graph. Users are free to construct the network using alternative potentials (for example, AMBER^69^ or CHARMM^70^) or using coarse-grained networks.

### Data availability

Data supporting this study (propensities and quantile scores for all 20 proteins in the test set) are available at figshare with DOI: 10.6084/m9.figshare.3413605.v1.

## Additional information

**How to cite this article:** Amor, B. R. C. *et al*. Prediction of allosteric sites and mediating interactions through bond-to-bond propensities. *Nat. Commun.* 7:12477 doi: 10.1038/ncomms12477 (2016).

## Supplementary Material

Supplementary Information Supplementary Figures 1-5, Supplementary Tables 1-6, Supplementary Notes 1-3, Supplementary Methods and Supplementary References.

## Figures and Tables

**Table 1:** Residue quantile scores of allosteric residues in caspase-1.

**Residue**	***p***_***R***_ **(Atomistic network)**		***p***_***R***_ **(RRIN)**	
	**Dimer 1**	**Dimer 2**	**Dimer 1**	**Dimer 2**
R240	0.772	0.734	0.562	0.562
L258	0.394	0.408	0.168	0.168
N259	0.828	0.832	0.324	0.324
F262	0.654	0.652	0.464	0.464
R286	0.938	0.928	0.838	0.838
C331	0.634	0.646	0.724	0.724
P335	0.206	0.196	0.450	0.450
E390	0.990	0.992	0.318	0.318
R391	0.982	0.984	0.258	0.258
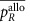	0.711	0.708	0.4567	0.4567
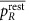	0.481	0.492	0.4793	0.4789

Quantile scores for the propensities of residues within 3.5 Å of the allosteric site of caspase-1 computed from the atomistic graph and from a residue-residue interaction network (RRIN) with cutoff radius of 6 Å. The average quantile scores of allosteric residues 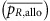 and non-allosteric residues 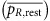 are also presented.

**Table 2:** Top residues by quantile score in CheY.

**Residue**	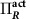	***p***_***R***_
D12	0.0076	1
E89*	0.0370	0.984
N62	0.0017	0.984
D57*	0.0094	0.968
K45	0.0015	0.968
T87*	0.0283	0.968
M85	0.0321	0.968
E35	0.0019	0.952
L116	0.0189	0.952
W58*	0.0247	0.936
L43	0.0030	0.921
F124	0.0120	0.905
L120	0.0189	0.905

Propensities of residues in CheY relative to the active site, ranked by quantile score (*p*_*R*_≥0.90). The star (*) indicates residues within 3.5 Å of the allosteric effector.

**Table 3:** Top bonds by quantile score in h-Ras.

**Bond**	**Π**_***b***_	**Distance (Å)**	***p***_***b***_
Q99:HOH727	0.0051	14.8	0.9991
K117:G13	0.026	2.76	0.9983
HOH727:S65	0.0067	12.2	0.9974
R164:E49	0.0013	25.0	0.9974
I21:S17	0.019	4.83	0.9965
D47:R161	0.0015	21.6	0.9948
H27:Q25	0.0075	10.8	0.9940
V8:L56	0.0010	9.05	0.9940
R161:D47	0.0013	21.6	0.9931
I24:K42	0.0035	14.8	0.9922
Q22:A146	0.017	5.09	0.9905

Top bonds ranked by propensity quantile score for h-Ras (*p*_*b*_≥0.99).
