# Supplementary material for: Prediction of allosteric sites and mediating interactions through bond-to-bond propensities
Source: Nat Commun. 2016 Aug 26;7:12477. doi: 10.1038/ncomms12477 (PMC5007447; doi:10.1038/ncomms12477)
Supplement: Supplementary Information — Supplementary Figures 1-5, Supplementary Tables 1-6, Supplementary Notes 1-3, Supplementary Methods and Supplementary References. [file ncomms12477-s1.pdf]

Supplementary Information

Supplementary Figures

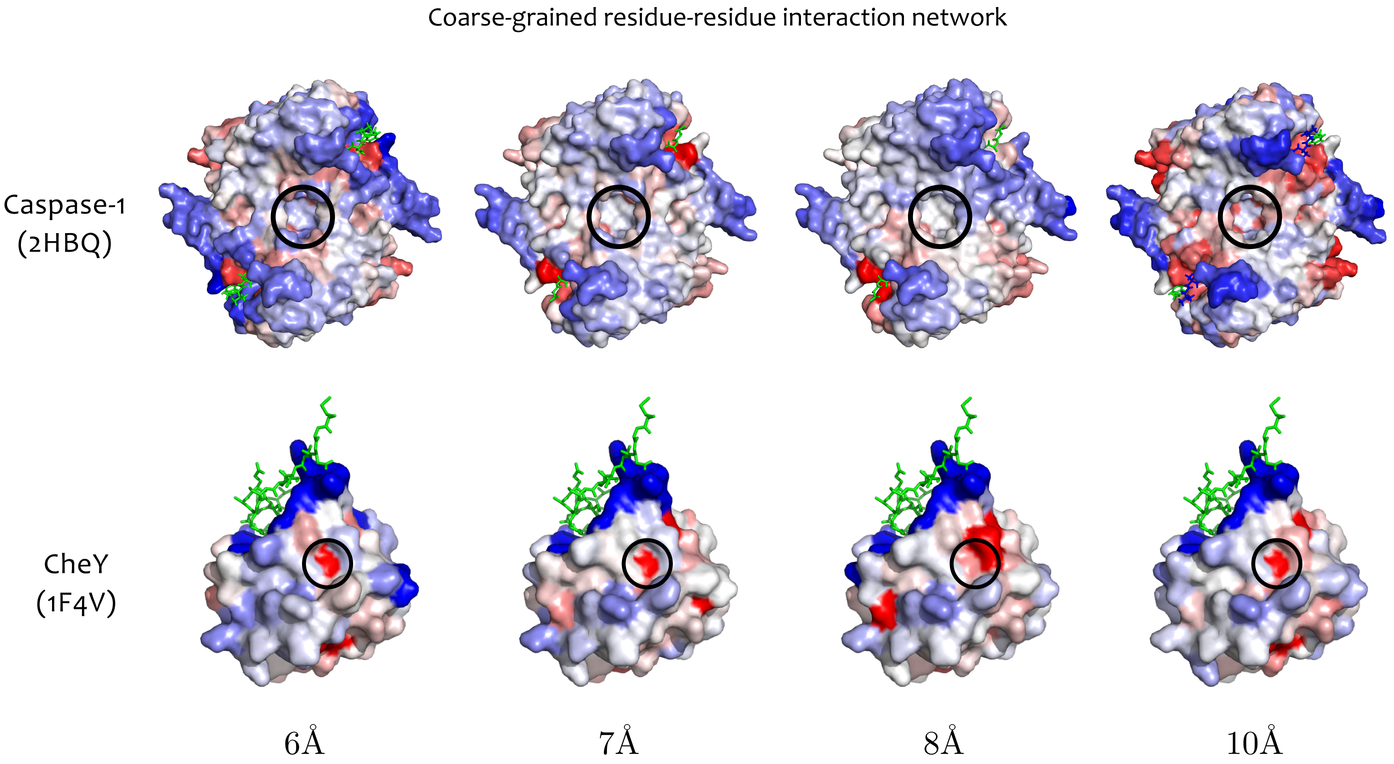

Supplementary Figure 1: **Quantile scores computed from RRINs for caspase-1 and CheY at different cut-off radii.** Surface mapping of the residue quantile scores p_R of caspase-1 and CheY for RRINs generated with radii cut-offs from 6 Å and 10 Å. The active-site ligand is shown in green sticks and the allosteric site is circled. The allosteric site in caspase-1 is not identified for 6, 7, and 8 Å. It is identified at 10 Å, but the signal is weaker than when using an atomistic graph. In contrast, for CheY the allosteric site is identified as significant across the full range of cut-offs.

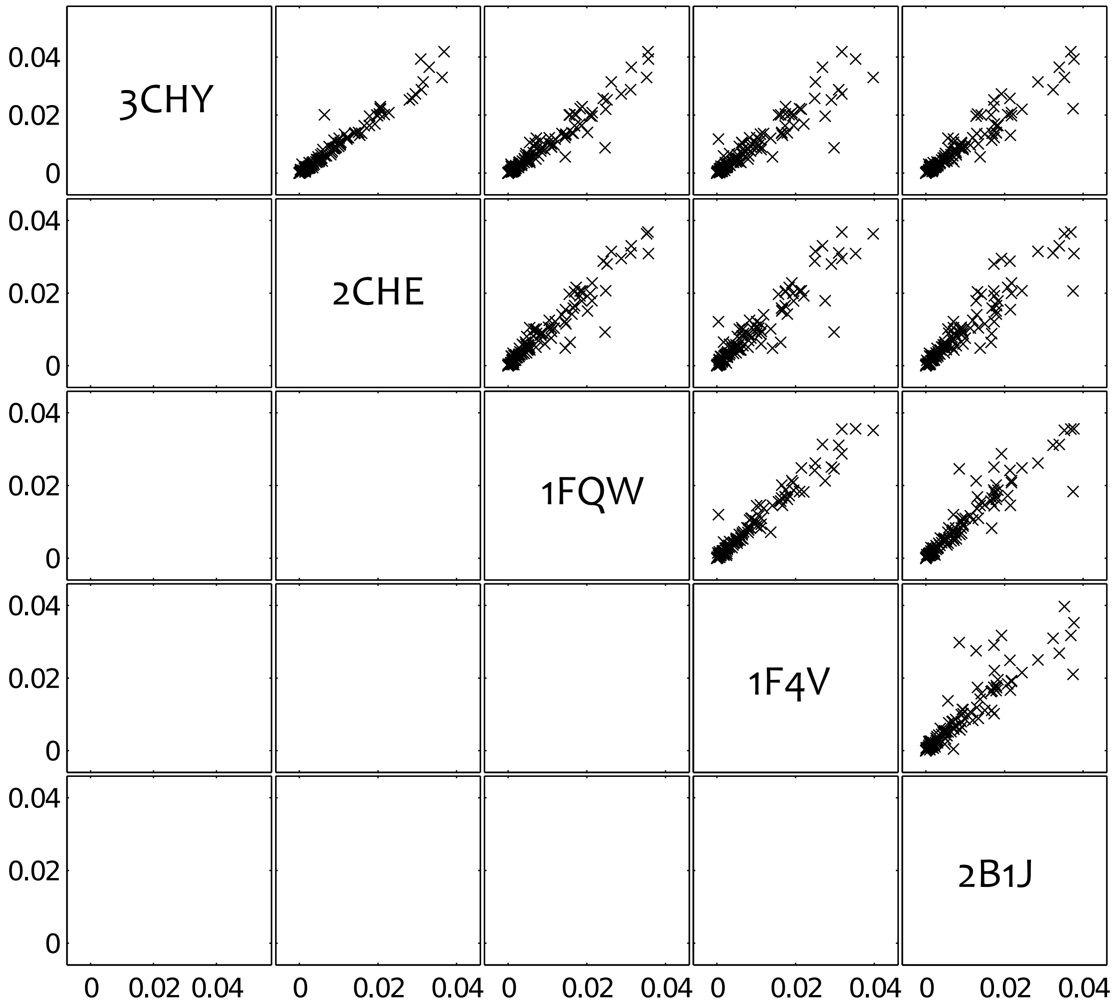

Supplementary Figure 2: **Propensities in different conformations of CheY.** Comparison of propensities of residues in across different structures of CheY: unbound (3CHY); bound to Mg^{2+} (2CHE); bound to Mn^{2+} and phosphate mimic BeFx (1FQW); bound to Mn^{2+}, BeFx and FliM (1F4V); and bound only to FliM (2B1J). The propensities of the residues are strongly correlated across states.

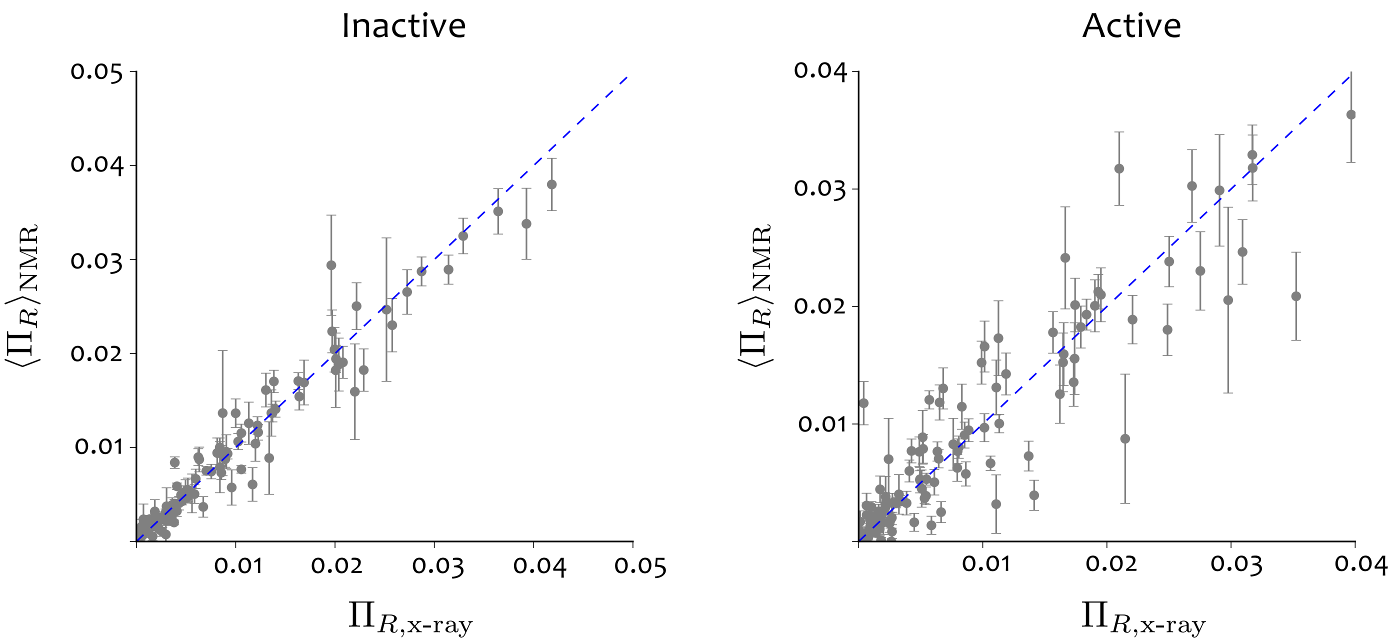

Supplementary Figure 3: **Propensities computed from CheY NMR ensembles.** Average propensity obtained from all structures in an NMR ensemble of CheY against the propensity obtained from the corresponding X-ray structure for inactive (left) and active (right). The inactive ensemble contains 20 structures and the active ensemble contains 27 structures. The error bars show the standard deviation of the propensities Π_R over the NMR ensemble. Both the variance and the deviation from the X-ray structure is greater for the active conformation.

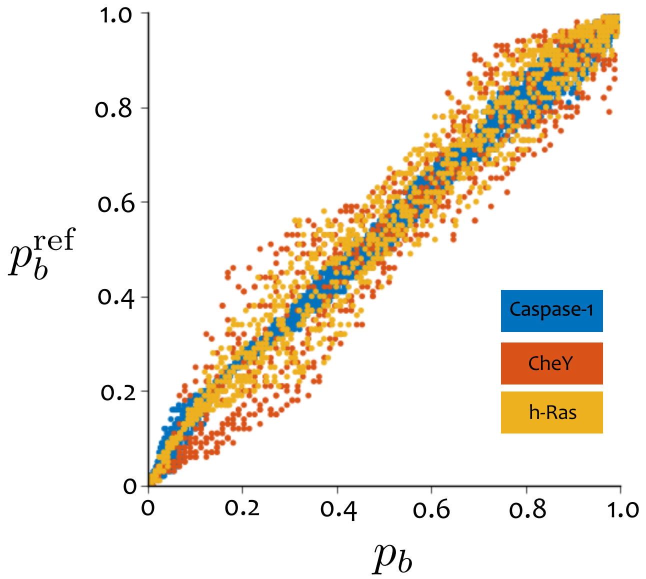

Supplementary Figure 4: **Absolute quantile scores versus intrinsic quantile scores.** The absolute quantile scores calculated from the reference set (p_b^{ref}) are plotted against the intrinsic quantile scores (p_b) for caspase-1 (blue), CheY (red), and h-Ras (yellow).

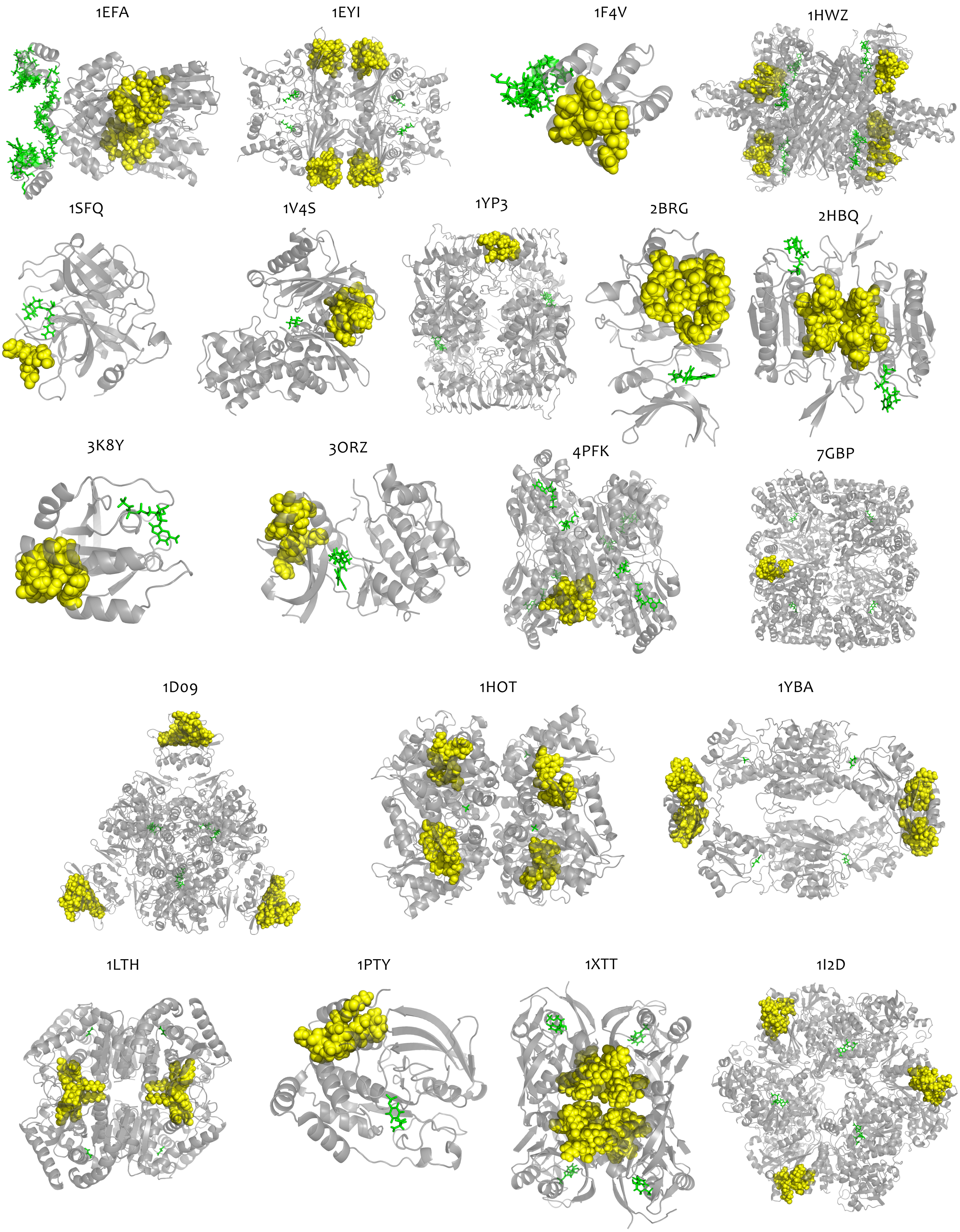

Supplementary Figure 5: **Allosteric test set.** The structures of the 20 proteins in the allosteric test set are shown with the active site ligand (green sticks) and allosteric site residues (yellow spheres).

Supplementary Tables

Supplementary Table 1: **Propensities computed from RRINs.** Values of $\overline{p_{R,\mathrm{allo}}} - \langle \overline{p_{R,\mathrm{site}}} \rangle_{\mathrm{surr}}$ for residue-residue interaction networks with four cut-off radii from 6Å–10Å. The propensity scores are shown in bold if they are greater than 0, and starred if they lie above the 95% confidence interval computed by a bootstrap with 10000 resamples. The comparable statistic computed from the all-atom network is also presented, as well as the summary of the four bond statistics for each protein from Supplementary Table 4.

		$\overline{p_{R,\mathrm{allo}}} - \langle \overline{p_{R,\mathrm{site}}} \rangle_{\mathrm{surr}}$				
PDB ID	All-atom summary	All-atom network	RRIN cut-off = 6Å	RRIN cut-off = 7Å	RRIN cut-off = 8Å	RRIN cut-off = 10Å
1V4S	●●●●	**0.35***	**0.065***	**0.010***	**0.047***	**0.16***
3ORZ	●●●●	**0.30***	**0.31***	**0.34***	**0.37***	**0.22***
1YP3	●●●●	**0.28***	-0.043	**0.11***	**0.046***	**0.13***
1D09	●●●●	**0.23***	**0.20***	**0.17***	**0.21***	**0.15***
2HBQ	●●●●	**0.15***	-0.079	-0.053	-0.062	**0.098***
1HOT	●●●●	**0.13***	-0.065	**0.13***	**0.18***	**0.20***
1PTY	●●○●	**0.11***	**0.11***	**0.088***	**0.050***	-0.032
1EYI	●●●○	**0.11***	-0.036	-0.081	-0.098	-0.029
7GPB	●●●○	**0.11***	**0.048***	**0.073***	**0.047***	-0.095
1F4V	●●●●	**0.096***	**0.14***	**0.11***	**0.23***	**0.071***
4PFK	●●●●	**0.092***	-0.13	-0.24	-0.19	-0.067
1I2D	●●●●	**0.091***	**0.034***	-0.091	**0.010***	**0.12***
1YBA	●●●●	**0.062***	**0.18***	**0.16***	**0.20***	**0.29***
1LTH	●●●●	**0.063***	**0.080***	-0.11	-0.22	-0.073
1XTT	○○●○	**0.0024**	**0.025***	-0.017	-0.012	**0.14***
1HWZ	○●●○	-0.015	**0.071***	**0.041***	-0.016	-0.0072
3K8Y	○○●○	-0.043	**0.29***	**0.24***	**0.17***	**0.30***
1EFA	○○○●	-0.066	-0.035	-0.0028	-0.064	-0.075
1SFQ	○●●●	-0.081	-0.18	-0.19	-0.16	-0.19
2BRG	○○○○	-0.24	**0.13***	-0.043	-0.057	-0.093

Supplementary Table 2: **Details of X-ray structures of CheY analysed.** The conformations correspond to different stages of activation.

PDB ID	Structural state	Resolution
3CHY	Unbound	1.7 Å
2CHE	Bound to Mg^{2+}	1.8 Å
1FQW	Bound to Mn^{2+} and BeFx	2.37 Å
2B1J	Bound to FliM	2.4 Å
1F4V	Bound to Mn^{2+}, BeFx and FliM	2.22 Å

Supplementary Table 3: **Proteins in the allosteric test set.** The active site and allosteric site bound structures for each of the 20 test set proteins. If the protein is allosterically activated then the PDB ID for both states will be the same. The ligand identifier is that used in the PDB file. Exceptions to this are CheY and caspase-1. As the ligand in these proteins is a peptide, the name and chain ID of the peptide is given instead.

		Active		Allosteric	
Protein	Residues	PDB	Ligand	PDB	Ligand
ATCase	2790	1D09	PAL	1RAC	CTP
Lac repressor	658	1EFA	NPF	1TLF	IPT
Fructose-1, 6-Bisphosphatase	1344	1EYI	F6P	1EYJ	AMP
CheY	144	1F4V	FliM (D)	1F4V	BEF
Glutamate DH	3018	1HWZ	NDP	1HWZ	GTP
ATP Sulfurylase	3444	1I2D	ADX	1M8P	PPS
PTP1B	299	1PTY	PTR	1T48	BB3
Thrombin	281	1SFQ	O6G	1SFQ	NA
Glucokinase	449	1V4S	GLC	1V4S	MRK
UPRTase	852	1XTT	U5P	1XTU	CTP
Phosphoglycerate DH	1644	1YBA	AKG	1PSD	SER451
ADP-glucose phosphorylase	1727	1YP3	ATP	1YP2	PMB
CHK1	258	2BRG	DFY	3JVS	AGY
Caspase-1	520	2HBQ	z-VAD-FMK (C/F)	2FQQ	F1G
PDK1	278	3ORZ	BI4	3ORZ	2A2
Phosphofructokinase	1288	4PFK	F6P	6PFK	PGA
Glycogen Phosphorylase	3304	7GPB	PLP/SO4	7GPB	SO4/AMP
glcN-6-P deaminase	1604	1HOT	PO4	1HOT	NAG/PHS
h-Ras	175	3K8Y	GNP	3K8Y	ACT
lactate DH	1260	1LTH	NAD	1LTH	FBP

Supplementary Table 4: **Allosteric site quantile scores in test set proteins.** The four scores described in Figure 7 of the main text for the test set of 20 proteins. The difference between the allosteric site average quantile score and the average surrogate site score for both residues and bonds are shown in bold if they are greater than 0, and starred if they lie above the 95% confidence interval computed by a bootstrap with 10000 resamples. The average reference quantile score $\overline{p^{\mathrm{ref}}_{b,\mathrm{allo}}}$ is shown in bold if it is greater than 0.5 (the expected value). The proportion $p_{b,\mathrm{allo}} > 0.95$ is shown in bold if it is greater than 0.05.

Protein	PDB ID	$\overline{p_{R,\mathrm{allo}}} - \langle\overline{p_{R,\mathrm{site}}}\rangle_{\mathrm{surr}}$	$\overline{p_{b,\mathrm{allo}}} - \langle\overline{p_{b,\mathrm{site}}}\rangle_{\mathrm{surr}}$	$\mathrm{P}(p_{b,\mathrm{allo}} > 0.95)$	$\overline{p^{\mathrm{ref}}_{b,\mathrm{allo}}}$	Summary
Glucokinase	1V4S	**0.35***	**0.14***	**0.12**	**0.66**	●●●●
PDK1	3ORZ	**0.30***	**0.030***	**0.080**	**0.56**	●●●●
ADP-glucose phosphorylase	1YP3	**0.28***	**0.074***	**0.10**	**0.59**	●●●●
ATCase	1DO9	**0.23***	**0.036***	**0.091**	**0.68**	●●●●
Caspase-1	2HBQ	**0.15***	**0.0032***	**0.070**	**0.54**	●●●●
glcN-6-P deaminase	1HOT	**0.13***	**0.031***	**0.079**	**0.51**	●●●●
PTP1B	1PTY	**0.11***	**0.0088***	0.048	**0.50**	●●○●
Fructose-1, 6-Bisphosphatase	1EYI	**0.11***	**0.033***	**0.052**	0.49	●●●○
Glycogen Phosphorylase	7GPB	**0.11***	**0.0027***	**0.058**	0.47	●●●○
Chemotaxis Y	1F4V	**0.096***	**0.055***	**0.074**	**0.58**	●●●●
Phosphofructokinase	4PFK	**0.092***	**0.068***	**0.16**	**0.54**	●●●●
ATP Sulfurylase	1I2D	**0.091***	**0.0313***	**0.068**	**0.52**	●●●●
Phosphoglycerate DH	1YBA	**0.062***	**0.076***	**0.075**	**0.59**	●●●●
Lactate DH	1LTH	**0.063***	**0.024***	**0.063**	**0.52**	●●●●
UPRtase	1XTT	**0.0024**	-0.013	**0.06**	0.44	○○●○
Glutamate DH	1HWZ	-0.015	**0.039***	**0.068**	0.44	○●●○
h-Ras	3K8Y	-0.043	-0.016	**0.059**	0.49	○○●○
Lac repressor	1EFA	-0.066	-0.016	0.014	**0.60**	○○○●
Thrombin	1SFQ	-0.081	**0.077***	**0.16**	**0.64**	○●●●
CHK1	2BRG	-0.24	-0.15	0.0052	0.36	○○○○

Supplementary Table 5: **Robustness of propensity scores to additive randomness.** Mean (± standard deviation) of propensity scores $\overline{p_{R,\text{allo}}} - \langle\overline{p_{R,\text{site}}}\rangle_{\text{surr}}$ computed from randomisations of the protein networks of the allosteric test set obtained by adding Gaussian noise to the edge weights (bond energies). The noise level varies between 1kT and 4kT (corresponding to the standard deviation of the added Gaussian) and at each noise level the results were calculated from 10 randomised graphs. The difference between the allosteric site average quantile score and the average surrogate site score for both residues and bonds are shown in bold if they are greater than 0, and starred if they lie above the 95% confidence interval computed by a bootstrap with 10000 resamples. The unperturbed result is also shown for comparison.

PDB ID	Unperturbed network	Gaussian noise 1kT	Gaussian noise 2kT	Gaussian noise 4kT
1V4S	**0.35***	**0.32±0.011***	**0.31±0.019***	**0.27±0.017***
3ORZ	**0.30***	**0.28±0.0087***	**0.23±0.0090***	**0.24±0.014***
1YP3	**0.28***	**0.27±0.0010***	**0.25±0.0088***	**0.17±0.016***
1D09	**0.23***	**0.22±0.0071***	**0.21±0.0024***	**0.20±0.0035***
2HBQ	**0.15***	**0.18±0.0096***	**0.20±0.0058***	**0.13±0.0097***
1HOT	**0.13***	**0.13±0.0061***	**0.098±0.024***	**0.12±0.021***
1PTY	**0.11***	**0.096±0.020***	**0.11±0.022***	**0.088±0.031***
1EYI	**0.11***	**0.13±0.0065***	**0.13±0.0022***	**0.16±0.0050***
7GPB	**0.11***	**0.096±0.018***	**0.13±0.010***	**0.14±0.015***
1F4V	**0.096***	**0.093±0.018***	**0.14±0.0097***	**0.12±0.027***
4PFK	**0.092***	**0.091±0.0052***	**0.11±0.022***	**0.12±0.0075***
1I2D	**0.091***	**0.14±0.029***	**0.14±0.030***	**0.14±0.037***
1YBA	**0.062***	**0.076±0.0034***	**0.091±0.0048***	**0.073±0.0051***
1LTH	**0.063***	**0.070±0.0099***	**0.063±0.016***	**0.039±0.019***
1XTT	**0.0024**	**0.0084±0.0069**	**0.015±0.0083**	**0.0077±0.0070**
1HWZ	-0.015	-0.0090±0.0071	-0.0028±0.0043	**0.011±0.0065**
3K8Y	-0.043	-0.033±0.012	-0.012±0.010	-0.025±0.022
1EFA	-0.066	-0.047±0.0054	-0.019±0.0078	-0.0027±0.0077
1SFQ	-0.081	-0.090±0.0089	-0.083±0.023	-0.10±0.028
2BRG	-0.24	-0.23±0.010	-0.24±0.013	-0.23±0.026

Supplementary Table 6: **Robustness of propensity scores to deletion of weak bonds.** The propensity score $\overline{p_{R,\text{allo}}} - \langle\overline{p_{R,\text{site}}}\rangle_{\text{surr}}$ for networks obtained by deleting all bonds below two energy thresholds. The results are shown in bold when they are greater than 0 and starred if they lie above the 95% confidence interval computed by a bootstrap with 10000 resamples. The unperturbed score is reported also for comparison.

PDB ID	Unperturbed network	Threshold 0.5 kT	Threshold 1kT
1V4S	**0.35***	**0.061***	**0.049***
3ORZ	**0.30***	**0.24***	**0.25***
1YP3	**0.28***	**0.24***	**0.30***
1D09	**0.23***	**0.088***	**0.10***
2HBQ	**0.15***	**0.16***	**0.18***
1HOT	**0.13***	**0.14***	**0.17***
1PTY	**0.11***	**0.13***	**0.080***
1EYI	**0.11***	**0.026***	**0.022***
7GPB	**0.11***	**0.056***	**0.062***
1F4V	**0.096***	-0.0010	**0.0085***
4PFK	**0.092***	**0.17***	**0.20***
1I2D	**0.091***	**0.0012**	-0.033
1YBA	**0.062***	**0.079***	**0.052***
1LTH	**0.063***	**0.056***	-0.081
1XTT	**0.0024**	-0.016	-0.023
1HWZ	-0.075	-0.016	-0.20
3K8Y	-0.043	-0.14	-0.16
1EFA	-0.066	**0.052***	**0.051***
1SFQ	-0.081	**0.073***	**0.11***
2BRG	-0.24	-0.20	-0.17

Supplementary Note 1

Propensities from residue-residue interaction networks

The computational efficiency of our methodology allows us to analyse all-atom networks without many of the restrictions on system size inherent to other methods. Proteins or protein complexes of hundreds of thousands of atoms can be analysed in a few minutes on a standard desktop. We can thus keep atomistic detail at the single bond level without restricting the scope of the analysis. Hence there is a less acute need to seek computational savings by obtaining coarse-grained representations of proteins at the level of residue interactions. However, it is still instructive to consider propensity measures computed from residue-level networks (RRINs) [1]. We have undertaken this comparison for all 20 proteins in our test set and report the results below.

As discussed in the main text (Sections IIA and IIB), in some cases (e.g., caspase-1, Fig 1b) we found that the additional information contained in the atomistic network leads to increased signal in the detection of the allosteric site, whereas in other cases (e.g., CheY), RRINs already capture well the site connectivity that reveals the presence of the allosteric site. Our analysis of the full test set (Supplementary Table 1) confirms that the results from RRINs depend on the protein analysed, and also vary substantially depending on the choice of the cut-off distance (a tunable parameter which needs to be chosen when generating the coarse-grained RRINs).

The coarse-grained RRINs for each of the 20 proteins in the test set were obtained by submitting the corresponding PDB files to the oGNM server [2]. We obtained RRINs at four different cut-off radii: 6 Å, 7 Å, 8 Å and 10 Å. The cut-off radius is a tunable parameter necessary to generate a RRIN from PDB files, which establishes how close two residues must be in order to be connected in the RRIN. A range of different cut-off radii has been used throughout the literature. However, the usual radius is around 6.7-7.0 Å, which corresponds to the first coordination shell [3].

Supplementary Table 1 shows the propensity score of the allosteric site $\overline{p_{R,\text{allo}}} - \langle \overline{p_{R,\text{site}}} \rangle_{\text{surr}}$, computed from RRINs obtained at four cut-offs (between 6Å and 10Å) for the 20 proteins in the allosteric test set. For comparison purposes, we also report the same score obtained from the all-atom network. It is important to note that this is just one of four scores obtained from the all-atom network, reflecting only the *averaged* behaviour over the residues. This score is complemented by the three other bond-based statistics, which can pick up inhomogeneities in the propensities of the bonds in the allosteric site, as given by the All-atom Summary column carried over from Supplementary Table 4.

Our results indicate broad consistency between RRINs and the all-atom network. However, the RRIN results vary widely depending on the choice of cut-off radius in the generation of the network. Moreover this variability with respect to the cut-off behaves differently for each of the proteins. As an illustration, the allosteric site of caspase-1 (2HBQ) was not found to be significant in the RRINs with cut-off radii of 6 Å, 7 Å and 8 Å, and only weakly significant for 10 Å, whereas 1LTH and 2BRG are both only detected in RRINS with cut-off radius of 6 Å but not for larger radii. Our results are consistent with previous studies that found that allosteric pathway identification in RRINs is dependent on the chosen cut-off [4]. For the different cut-offs, the number of proteins with $p_{R,\text{allo}} > p_{R,\text{rest}}$ varies between 11/20 (at 7, 8, and 10Å) and 13/20 (at 6Å), and only 8/20 proteins have $p_{R,\text{allo}} > p_{R,\text{rest}}$ for the RRINS at *all* the cut-off radii. This is compared to 15/20 proteins for the atomistic network.

Even when the allosteric site is detected in the RRIN, the signal when using the atomistic network is considerably higher in a number of proteins (e.g., 1V4S, 1YP3, 7GPB, 1I2D, 2HBQ). In other cases (e.g., 1EYI, 4PFK), the RRIN directly loses the detectability of the allosteric site even if the cut-off is adjusted. This observation suggests that these are proteins where the specific chemistry of intra-protein bonds is important for the allosteric communication.

On the other hand, there are several other cases (e.g., 3ORZ, 1D09, 1HOT, 1PTY, 1LTH) where the RRIN can provide similar results to the atomistic network, yet still with some variability depending on the choice of appropriate cut-off. Interestingly, there are also some proteins (specifically 1F4V, 1YBA, 3K8Y and 2BRG) in which the propensity score is higher for RRINs than for the atomistic network. In these cases, there tends to be a large heterogeneity in the propensities of the bonds in the allosteric site (see Figure 7 in the main text) with some bonds with large negative values as well as other bonds with large positive values. Our bond statistical measures can account for some of this variability. Indeed, both 1F4V and 1YBA are detected by all our four bond measures, and 3K8Y is picked by the measure based on the distributions of p_b. Intriguingly, only 2BRG (corresponding to CHK1) cannot be detected by our bond measures. This suggests other areas of future research, in which the importance of averaging at the level of pathways could be used to enrich the findings presented here.

Supplementary Note 2

Robustness of the bond-to-bond propensities to random perturbations of the weak interactions

Proteins are dynamic objects undergoing motions and fluctuations under the influence of the environment. Such dynamic fluctuations induce changes in the bond energies of the protein, potentially leading to the breaking of weak bonds (hydrogen bonds, salt bridges, hydrophobic tethers). As discussed in the main text when studying the NMR ensemble of conformations of CheY (Section IIB.3), whilst there is considerable agreement between the results from the NMR structures and the X-ray structure (Fig. 3), the variability in the ensemble can reveal further information. It is also important to check that the computation of propensities is generally robust to the presence of such noise. To do this, we have developed two schemes to add random perturbations to our protein networks. These schemes mimic the effect of small dynamic fluctuations, without carrying out expensive molecular dynamics simulations.

Firstly, for each of the 20 proteins in our dataset, we add zero mean Gaussian noise to the edge weights (energies) of non-covalent bonds in the graph, so as to mimic the effect of thermal fluctuations. Note that we allow the bonds to break if their randomised energy becomes zero. We then recompute our quantile scores for the allosteric site for 10 realisations of the noisy networks generated after the addition of the Gaussian fluctuations. We do this for 3 levels of noise, i.e., we increase the standard deviation of the Gaussian from 1kT=0.6 kcal/mol to 4kT=2.4 kcal/mol. The average results of these randomisations for all proteins in the allosteric test set are presented in Supplementary Table 5. Our calculations show that the results are generally robust to fluctuations induced in this way: the signal at the allosteric site only drops slightly when introducing relatively high levels of noise.

Secondly, to test a different kind of variability introduced by the environment, we have considered the effect of breaking all bonds in our network with energy below a threshold. Starting with the original unperturbed structure, all weak bonds below a given threshold are removed from the graph. In this way, we mimic the possibility of extended structural changes that could lead to breaking of bonds in a more global fashion.

For each of the 20 proteins in the test set, we generate two perturbed networks obtained by bond removal of *all* bonds with energy below two different thresholds: 0.5 kT$\simeq$ 0.3 kcal/mol and 1kT $\simeq$ 0.6 kcal/mol. The effect of this thresholding is extensive. For the 0.5kT threshold, we delete all hydrophobic tethers and electrostatic interactions as well as a percentage of hydrogen bonds that ranges from 31% in 1SFQ to 44% in 1HWZ and 1LTH. For the 1kT threshold, even further hydrogen bonds are removed, corresponding to eliminating 44% of H-bonds in 1SFQ up to 57% of the H-bonds in 7GPB, 2BRG, 1LTH (in addition to all hydrophobic interactions).

The calculations of the propensity for the thresholded networks for all 20 proteins in our test set are presented in Supplementary Table 6. Our results show that, overall, the propensity of the allosteric site remains largely robust to such changes across all 20 proteins considered, yet with notable differences in the magnitude of the effect across the set. In some proteins, the signal at the allosteric site is mildly affected by bond deletion (e.g. 3ORZ, 1YP3, 2HBQ, 1HOT, 1PTY). In other cases, however, the deletion of weaker hydrogen bonds has a large effect in destroying the communication between the allosteric site and the active site (e.g. 1V4S, 1D09, 1EYI, 7GPB, 1F4V). These differences could be a measure of how robust the allosteric signalling is to energetic fluctuations in the local environment of the protein, and also provide clues as to different structural features connected with the distributed nature of allosteric signalling in the different proteins. The study of such differences will be the object of future work.

Supplementary Note 3

Derivation of the graph-theoretical formula for edge fluctuations

We now derive in more detail Eq.(5), presented in Materials and Methods (Section IVA) in the main text. Let us consider the Langevin equation, Eq.(1) in the main text:

$$\dot{\mathbf{x}} = -L\mathbf{x} + \boldsymbol{\epsilon}, \tag{1}$$

where $\boldsymbol{\epsilon}$ is white Gaussian noise. Without loss of generality, we may assume that the system started initially from a condition $\mathbf{x}(-\infty) = 0$. A standard result from linear system theory is that the solution of equation (1) is given by:

$$\mathbf{X}(t) = \int_{-\infty}^{t} \exp[-L(t-s)]\boldsymbol{\epsilon}(s)ds. \tag{2}$$

Since our input $\boldsymbol{\epsilon}$ is random, $\mathbf{X}(t)$ is a random process, which we indicate by the upper-case notation. Likewise, the edge variables will be described by the random process:

$$\mathbf{Y}(t) = B^T \int_{-\infty}^{t} \exp[-L(t-s)]\boldsymbol{\epsilon}(s)ds, \tag{3}$$

where B is the incidence matrix of the graph of the protein.

The autocorrelation of the process $\mathbf{Y}(t)$ for $\tau > 0$ is then

$$\begin{aligned}
\mathcal{R}(\tau) &= \mathbb{E}[\mathbf{Y}(t)\mathbf{Y}^T(t+\tau)] = \mathbb{E}\left[\int_{-\infty}^{t+\tau}\int_{-\infty}^{t} B^T \exp[-L(t-s)]\boldsymbol{\epsilon}(s)\boldsymbol{\epsilon}(\xi)^T \exp[-L(t+\tau-\xi)]^T B\, ds\, d\xi\right] \\
&= \int_{-\infty}^{t+\tau}\int_{-\infty}^{t} B^T \exp[-L(t-s)]\mathbb{E}\left[\boldsymbol{\epsilon}(s)\boldsymbol{\epsilon}(\xi)^T\right] \exp[-L(t+\tau-\xi)]^T B\, ds\, d\xi \\
&= \int_{-\infty}^{t+\tau}\int_{-\infty}^{t} B^T \exp[-L(t-s)]\left[\delta(s-\xi)\, I\right] \exp[-L(t+\tau-\xi)]^T B\, ds\, d\xi \\
&= \int_{-\infty}^{t} B^T \exp[-L(t-\xi)] \exp[-L(t+\tau-\xi)]^T B\, d\xi = \int_{-\infty}^{t} B^T \exp[-L(2t-2\xi+\tau)]B\, d\xi,
\end{aligned} \tag{4}$$

where we have used the fact that the noise vector $\boldsymbol{\epsilon}$ is delta-correlated in time and across nodes (i.e., $I = \delta_{ij}$ is the identity matrix). The last equality follows from fact that $L = L^T$; hence it commutes and this implies that $\exp(Lt)\exp(Lt)^T = \exp(2Lt)$.

This integral can be computed using the eigendecomposition of the matrix exponential as follows:

$$\begin{aligned}
\mathcal{R}(\tau) &= \int_{-\infty}^{t} B^T \exp[-L(2t-2\xi+\tau)]B\, d\xi = \sum_{i=1}^{N}\int_{-\infty}^{t} B^T e^{-\lambda_i(2t-2\xi+\tau)}\mathbf{v}_i\mathbf{v}_i^T B\, d\xi \\
&= \frac{1}{N}\int_{-\infty}^{t} B^T \mathbf{1}\mathbf{1}^T B\, d\xi + \sum_{i=2}^{N}\int_{-\infty}^{t} B^T e^{-\lambda_i(2t-2\xi+\tau)}\mathbf{v}_i\mathbf{v}_i^T B\, d\xi = \sum_{i=2}^{N}\int_{-\infty}^{t} B^T e^{-\lambda_i(2t-2\xi+\tau)}\mathbf{v}_i\mathbf{v}_i^T B\, d\xi \\
&= B^T\left[\sum_{i=2}^{N} \left.\frac{e^{-\lambda_i(\tau+2t-2\xi)}}{2\lambda_i}\right|_{\xi=-\infty}^{t} \mathbf{v}_i\mathbf{v}_i^T\right] B = \frac{1}{2}B^T\left[\sum_{i=2}^{N}\frac{1}{\lambda_i}e^{-\lambda_i\tau}\mathbf{v}_i\mathbf{v}_i^T\right] B \\
&= \frac{1}{2}B^T\left[\sum_{i=2}^{N}\frac{1}{\lambda_i}\mathbf{v}_i\mathbf{v}_i^T\sum_{j=1}^{N}e^{-\lambda_j\tau}\mathbf{v}_j\mathbf{v}_j^T\right] B = \frac{1}{2}B^T L^{\dagger}\exp(-\tau L)B.
\end{aligned} \tag{5}$$

Here we have used the fact that the leading eigenvector of L associated with $\lambda_1 = 0$ is the vector of ones ($\mathbf{v}_1 = \mathbf{1}$), which is in the null space of B^T, i.e., $B^T\mathbf{1} = 0$. In the last two equations we have made use of the orthonormality of the eigenvectors ($\mathbf{v}_i^T\mathbf{v}_j = \delta_{ij}$), which implies that $\mathbf{v}_i\mathbf{v}_i^T = \mathbf{v}_i\mathbf{v}_i^T\sum_{j=1}^{N}\mathbf{v}_j\mathbf{v}_j^T$.

Supplementary Methods

1 Propensities of CheY conformations: different activation states and NMR ensemble

1.1 Active and inactive conformations of CheY

We calculated the propensities of residues for several CheY structures representing different activation states. Details of the different structures are given in Supplementary Table 2 and a comparison of the perturbation propensities across the different structures is shown in Supplementary Figure 2. As discussed in Section IIB.2, the propensities of the residues are strongly correlated across states. In the main text (Section IIB.2 and Figure 3), we concentrate on the comparison of 1F4V (active) against 3CHY (inactive).

1.2 CheY structures from NMR experiments

We also calculated the perturbation propensities of residues across two ensembles of NMR structures for active CheY (PDB ID: 1DJM; 27 structures) and inactive CheY (PDB ID: 1CYE; 20 structures). A comparison of the average propensity of each residue (averaged across the NMR ensemble) versus its propensity in the X-ray structure is shown in Supplementary Figure 3 for both the active ensemble (1DJM) and the inactive ensemble (1CYE). This data is discussed in the main text (Section IIB.3 and Figure 4).

2 The protein reference set from the SCOP database and absolute quantile scores

SCOP database: We have collected a random reference set of 100 proteins from the Structural Classification of Proteins (SCOP) database [5]. Protein domains in the SCOP database are classified according to a hierarchy based on structural similarity. Although proteins are additionally divided into superfamilies and subfamilies according to structural and sequence similarity, the major classes are:

1. All α: protein domains containing only alpha-helices
2. All β: protein domains containing only beta-sheets
3. Alpha and beta (α/β): protein domains containing both α-helices and β-sheets, with mainly parallel β-sheets.
4. Alpha and beta (α+β): protein domains containing both α-helices and β-sheets, with mainly anti-parallel β-sheets.
5. Multi-domain: folds of two or more domains from different classes.

We chose 20 proteins from each of these five classes uniformly at random from all proteins in each class, yet choosing only from structures where there is a ligand bound to the active site.

Absolute quantile scores: On this set of 100 proteins, we then identified the active site in each protein and computed the propensity for all its bonds relative to the active site. Across the set of 100 proteins in the reference set, we have a total of 465,409 non-covalent bonds, on which we apply quantile regression to obtain absolute quantile scores p^{ref}. In Supplementary Figure 4, the quantile scores p_b for all the bonds of the three proteins studied in detail in the main text (caspase-1, Che-Y, h-Ras) are plotted against their absolute quantile score p_b^{ref}, showing a good correlation overall. In general, we observe a tighter correlation for larger proteins (e.g., caspase-1), as a result of the QR fit being based on the number of bonds, E, which is related to the size of the protein.

3 Bond-to-bond propensities of the allosteric test set

3.1 Description of the allosteric test set

As discussed in the main text (Section IIE), we have constructed a test set of 20 allosteric proteins on which to benchmark our algorithm. Each protein in our test set has a structure with a bound active site ligand and a structure with a bound allosteric ligand. If the protein is allosterically activated then we use a single structure in which the protein is complexed with both the activator and the active site ligand. Ref. [6] collected a test set of 15 allosteric proteins for which both active site bound and allosteric site bound structures are available. We have used 10 of these proteins (the other five were found to be unsuitable for our analysis due to the presence of many non-standard amino-acids, mismatch between the oligomeric state of the active and inactive structures, or the absence of an allosteric ligand). We have enlarged the set with a further 10 proteins from an extensive search of the literature. The structures of the 20 proteins are shown in Supplementary Figure 5, with the active site indicated by the green ligand, and the allosteric site indicated by the yellow spheres. The allosteric site is defined as any residue containing an atom within 4Å of the allosteric ligand; allosteric site

bonds are defined as any weak interactions formed by an allosteric residue. Full details of the proteins and allosteric site residues are shown in Supplementary Table 3.

3.2 Summary of results on the allosteric test set

As explained in the main text (Section IIE and Materials and Methods, Section IVD), for each of the 20 proteins in the test set, we analyse the propensities of all bonds with respect to the active site of the bound structure, using the ligands shown in Fig. 5 as the source for the bond-to-bond propensity calculations. For each protein, we obtain the propensity Π_b of every weak bond and its associated quantile score (p_b). To establish their statistical significance, the bond quantile scores p_b (and residue averaged quantile scores p_R) of the allosteric site are compared against an ensemble of randomly generated surrogate sites from each protein. The ensemble of surrogate sites is constructed at random by picking sites that satisfy two structural constraints: (i) they have the same number of residues as the allosteric site; and (ii) their diameter (the maximum distance between any two atoms in the site) is no larger than that of the allosteric site. The sites are generated using Algorithm 1 with pseudocode given below.

Algorithm 1 Pseudocode for surrogate site sampling

site $\leftarrow \varnothing$
while # residues in site < # residues in allosteric site **do**
    choose a residue R at random
    if diameter(site $\cup$ R) < diameter(allosteric site) **then**
        site $\leftarrow$ site $\cup$ R
    end if
end while

The propensities averaged over the ensemble of surrogate sites are then used for statistical comparison with the allosteric site. We also obtain absolute propensity scores for each bond (p_b^{ref}) by comparing against the reference SCOP ensemble of 100 proteins. These quantities are defined in the main text (Materials and Methods, Section IVD). Using all these scores we obtain our four statistical measures of significance summarised in Supplementary Table 4. These numerical results are presented also in the form of a graph in Figure 7 of the main text.

4 Construction of the atomistic protein network

As discussed in Materials and methods (Section IVE), the protein network is constructed by assigning edges between atoms which interact covalently and non-covalently. Each edge is weighted by the strength of the interaction. Covalent bond strengths are obtained from tables assuming standard bond lengths. We include three types of non-covalent interactions: hydrophobic interactions, hydrogen bonds, and electrostatic interactions. The assignment of bonds in the graph follows from the well established FIRST framework [7, 8]. More in detail:

- **Covalent bonds:** Covalent bonds are weighted according to standard bond dissociation energies given in Ref. [9].
- **Hydrophobic tethers:** Hydrophobic tethers are assigned between C-C or C-S pairs based on proximity: two atoms have a hydrophobic tether if their Van der Waals' radii are within 2 Å. The hydrophobic tethers are identified using FIRST [10], which does not assign them an energy, and the energy is then determined based on the double-well potential of mean force introduced by Lin *et al* [11], which gives an energy of ≈ -0.8kcal/mol for atoms within 2 Å.
- **Hydrogen bonds:** The energies of hydrogen bonds were calculated using the same formula used by the program FIRST [10] and is based on the potential introduced by Mayo *et al* [12].
- **Electrostatic interactions:** Important electrostatic interactions between ions and ligands, as defined in the LINK entries of the PDB file, are added with energies derived from a Coulomb potential

$$E_{\mathrm{Coul}} = \frac{332}{\epsilon}\frac{q_1 q_2}{r}, \tag{6}$$

  where q_1 and q_2 are the atom charges, r is the distance between them, and ϵ is the dielectric constant, which is set to $\epsilon = 4$ as in Ref. [13]. Atom charges for standard residues are obtained from the OPLS-AA force field [14], whereas charges for ligands and non-standard residues are found using the PRODRG web-server [15].

An extended discussion of the construction of the atomistic graph can be found in Refs. [16, 17, 18]

Supplementary References

[1] C. Chennubhotla and I. Bahar, "Signal propagation in proteins and relation to equilibrium fluctuations," *PLoS computational biology*, vol. 3, no. 9, p. e172, 2007.

[2] L.-W. Yang, A. Rader, X. Liu, C. J. Jursa, S. C. Chen, H. A. Karimi, and I. Bahar, "oGNM: online computation of structural dynamics using the Gaussian Network Model," *Nucleic acids research*, vol. 34, no. suppl 2, pp. W24–W31, 2006.

[3] A. R. Atilgan, P. Akan, and C. Baysal, "Small-world communication of residues and significance for protein dynamics," *Biophysical journal*, vol. 86, no. 1, pp. 85–91, 2004.

[4] A. A. Ribeiro and V. Ortiz, "Determination of signaling pathways in proteins through network theory: importance of the topology," *Journal of Chemical Theory and Computation*, vol. 10, no. 4, pp. 1762–1769, 2014.

[5] L. L. Conte, B. Ailey, T. J. Hubbard, S. E. Brenner, A. G. Murzin, and C. Chothia, "SCOP: a structural classification of proteins database," *Nucleic acids research*, vol. 28, no. 1, pp. 257–259, 2000.

[6] M. Daily, T. Upadhyaya, and J. Gray, "Contact rearrangements form coupled networks from local motions in allosteric proteins," *Proteins: Structure, Function, and Bioinformatics*, vol. 71, no. 1, pp. 455–466, 2008.

[7] D. J. Jacobs and M. F. Thorpe, "Generic rigidity percolation: the pebble game," *Physical review letters*, vol. 75, no. 22, p. 4051, 1995.

[8] D. J. Jacobs and M. F. Thorpe, "Computer-implemented system for analyzing rigidity of substructures within a macromolecule," Jan. 11 1999. US Patent 6,014,449.

[9] J. Huheey, E. Keitler, and R. Keitler, *Inorganic Chemistry, Principles of Structure and Bonding.* Harper Collins College Publishers, New York, 1993.

[10] D. Jacobs, A. Rader, L. Kuhn, and M. Thorpe, "Protein flexibility predictions using graph theory," *Proteins: Structure, Function, and Bioinformatics*, vol. 44, no. 2, pp. 150–165, 2001.

[11] M. S. Lin, N. L. Fawzi, and T. Head-Gordon, "Hydrophobic potential of mean force as a solvation function for protein structure prediction," *Structure*, vol. 15, no. 6, pp. 727–740, 2007.

[12] B. Dahiyat, D. Benjamin Gordon, and S. Mayo, "Automated design of the surface positions of protein helices," *Protein Science*, vol. 6, no. 6, pp. 1333–1337, 1997.

[13] M. K. Gilson and B. H. Honig, "The dielectric constant of a folded protein," *Biopolymers*, vol. 25, no. 11, pp. 2097–2119, 1986.

[14] W. L. Jorgensen and J. Tirado-Rives, "The OPLS [optimized potentials for liquid simulations] potential functions for proteins, energy minimizations for crystals of cyclic peptides and crambin," *Journal of the American Chemical Society*, vol. 110, no. 6, pp. 1657–1666, 1988.

[15] A. W. Schuèttelkopf and D. M. Van Aalten, "PRODRG: a tool for high-throughput crystallography of protein–ligand complexes," *Acta Crystallographica Section D: Biological Crystallography*, vol. 60, no. 8, pp. 1355–1363, 2004.

[16] A. Delmotte, E. Tate, S. Yaliraki, and M. Barahona, "Protein multi-scale organization through graph partitioning and robustness analysis: application to the myosin–myosin light chain interaction," *Physical Biology*, vol. 8, p. 055010, 2011.

[17] A. Delmotte, *All-scale structural analysis of biomolecules through dynamical graph partitioning.* PhD thesis, Imperial College London, 2014.

[18] B. Amor, S. Yaliraki, R. Woscholski, and M. Barahona, "Uncovering allosteric pathways in caspase-1 using Markov transient analysis and multiscale community detection," *Molecular BioSystems*, vol. 10, no. 8, pp. 2247–2258, 2014.
